# Genome-wide identification and characterization of *NBLRR* genes in finger millet (*Eleusine coracana* L.) and their expression in response to *Magnaporthe grisea* infection

**DOI:** 10.1186/s12870-024-04743-z

**Published:** 2024-01-29

**Authors:** Alexander Balamurugan, Mallana Gowdra Mallikarjuna, Shilpi Bansal, S. Chandra Nayaka, Hosahatti Rajashekara, Tara Satyavathi Chellapilla, Ganesan Prakash

**Affiliations:** 1 Division of Plant Pathology, ICAR-Indian Agricultural Research Institute, New Delhi, 110012 India; 2 Division of Genetics, ICAR-Indian Agricultural Research Institute, New Delhi, 110012 India; 3 Department of Studies in Applied Botany and Biotechnology, University of Mysore, Mysore, 570005 India; 4 ICAR-Vivekananda Institute of Hill Agriculture, Almora, Uttarakhand, 263601 India; 5 ICAR-Indian Institute of Millets Research, Rajendranagar, Hyderabad, Telangana 500 030 India; 6 Present Address: Department of Science and Humanities, SRM Institute of Science and Technology, Modinagar, Uttar Pradesh 201204 India

**Keywords:** Blast, evolution, Finger millet, Expression analysis, *Magnaporthe Grisea*, *NBLRR*

## Abstract

**Background:**

The nucleotide binding site leucine rich repeat (NBLRR) genes significantly regulate defences against phytopathogens in plants. The genome-wide identification and analysis of *NBLRR* genes have been performed in several species. However, the detailed evolution, structure, expression of *NBLRR*s and functional response to *Magnaporthe grisea* are unknown in finger millet (*Eleusine coracana* (L.) Gaertn.).

**Results:**

The genome-wide scanning of the finger millet genome resulted in 116 *NBLRR* (*EcNBLRRs1-116*) encompassing 64 CC-NB-LRR, 47 NB-LRR and 5 CC_R_-NB-LRR types. The evolutionary studies among the NBLRRs of five Gramineae species, *viz.,* purple false brome (*Brachypodium distachyon* (L.) P.Beauv.), finger millet (*E. coracana*), rice (*Oryza sativa* L.), sorghum (*Sorghum bicolor* L. (Moench)) and foxtail millet (*Setaria italica* (L.) P.Beauv.) showed the evolution of NBLRRs in the ancestral lineage of the target species and subsequent divergence through gene-loss events. The purifying selection (Ka/Ks < 1) shaped the expansions of *NBLRRs* paralogs in finger millet and orthologs among the target Gramineae species. The promoter sequence analysis showed various stress- and phytohormone-responsive *cis*-acting elements besides growth and development, indicating their potential role in disease defence and regulatory mechanisms. The expression analysis of 22 *EcNBLRRs* in the genotypes showing contrasting responses to *Magnaporthe grisea* infection revealed four and five *EcNBLRRs* in early and late infection stages, respectively. The six of these nine candidate EcNBLRRs proteins, *viz*., EcNBLRR21, EcNBLRR26, EcNBLRR30, EcNBLRR45, EcNBLRR55 and EcNBLRR76 showed CC, NB and LRR domains, whereas the EcNBLRR23, EcNBLRR32 and EcNBLRR83 showed NB and LRR somains.

**Conclusion:**

The identification and expression analysis of *EcNBLRRs* showed the role of *EcNBLRR* genes in assigning blast resistance in finger millet. These results pave the foundation for in-depth and targeted functional analysis of *EcNBLRRs* through genome editing and transgenic approaches.

**Supplementary Information:**

The online version contains supplementary material available at 10.1186/s12870-024-04743-z.

## Background

Millets are one of the earliest domesticated small-seeded annual grass crops. Millets make up an important portion of the food basket in ~130 countries, which serves as a traditional food source for more than 590 million people in Asia and Africa. Among various millets used as food crops, the finger millet (*Eleusine coracana* L. Gaertn.) is considered one of the most important millets owing to wider cultivation in rainfed areas, its ability to sustain harsh dry environments and low soil fertility and its utility as a source of nutrition in poverty-stricken arid and semi-arid regions [1–3]. Since finger millet grains contain a significant portion of fibres, protein, vitamin B, minerals, essential amino acids, calcium and iron; hence, it is considered nutritionally superior to wheat, rice and maize [1, 4, 5]. Apart from human consumption, the finger millet straw is used as animal fodder, which possesses 60% of digestible nutrients [6]. The global finger millet production of 4.5 million tons (https://www.fao.org/faostat/en/) is insufficient to meet the current and increasing population demand. On the other hand, finger millet production and productivity are constrained by various stresses,* viz.,* pests, diseases, drought, low nutrition, etc. [7]. Among these production constraints, the finger millet blast caused by an ascomycete filamentous fungus, *Magnaporthe grisea* (anamorphic stage: *Pyricularia grisea*), is the most devastating foliar pathogen that heavily affects the production and productivity of finger millet [8] and causes substantial yield losses up to 50–100% [7]. Among the various available approaches, managing finger millet blast by exploiting host-pathogen interaction-based genetic resistance mechanisms is the most sustainable, eco-friendly and farmer-friendly approach.

The host has a genetically imprinted innate immune system that resists the pathogen attack mainly in the form of pathogen-associated molecular patterns (PAMP)-triggered immunity (PTI) and effectors-triggered immunity (ETI) [9]. Firstly, PTI will activate when the molecules from the pathogen interact with extracellular pattern-recognition receptors (PRR) lined up in the host plasma membrane [10, 11]. Nevertheless, to counteract the PTI, some pathogens secrete the effector or avirulent (*Avr*) proteins into the plant cell and suppress the PTI, leading to infection. The secondary defence mechanism of ETI will be functioning where cytoplasmic immune receptors called R-proteins (resistance) recognize pathogen-derived *Avr* proteins, leading to a cascade of defence signalling pathways resulting in hypersensitive reaction (HR) or localized programmed cell death (PCD) [9, 12, 13]. Numerous *R* genes have been isolated in the last few years, especially from ice [14], cottonwood [15], papaya [16], *Arabidopsis thaliana* [17] and *Brassica rapa* [18] through genome-wide mining and characterization. The *NBLRR* (nucleotide-binding sites leucine rich repeats) shows the NBS domain at the amino-terminal or central and LRR domain with leucine and hydrophobic amino acids towards the C terminal [13, 19, 20] constitute most of the existing R proteins in plants. The *NBLRR* genes encompass approximately 0.2–1.6% of the genome of plant species [21]. The NBS domain is involved in signalling through interaction with bound ATP and GTP, whereas the C-terminal LRR domain helps in the recognition of pathogens as well as protein-protein interactions in establishing disease resistance reactions [22, 23]. The role of *NBLRR* genes in disease resistance was reported in various crops, including powdery mildew in sunflower [24], various pathogens in cotton [25] and yam [26], powdery mildew in *Vitis vinifera* [13], downy mildew and black rot in Chinese cabbage [27] etc. The comparative genomic analysis showed homology between *NBLRR* and *EST* (expressed sequence tags) sequences of finger millet and *Pi21* and *Pikh* of rice, which indicated conserved orthology of blast resistance among finger millet and rice lineage [28, 29].

There were few efforts on the evolutionary and functional characterization of *NBLRR* genes in finger millet [30, 31]. However, these studies primarily relied on searching the NCBI nucleotide sequence archives with search terms and PCR-based gene mining using degenerative primers. They could not capture the complete genome-wide snapshot of finger millet *NBLRR*s. Secondly, there was no in-depth evolutionary analysis among the NBLRRs of finger millet and other grass species. Thus, we first framed our investigation to identify the finger millet NBLRR genes in the latest finger millet genome using homology- and HMM-based approaches. Secondly, our emphasis is to comprehensively study the evolution and functional response of *NBLRR* genes to *Magnaporthe grisea* infection in finger millet.

## Results

### Mining and physicochemical characterization of NBLRR sequences

**Fig. 1:**
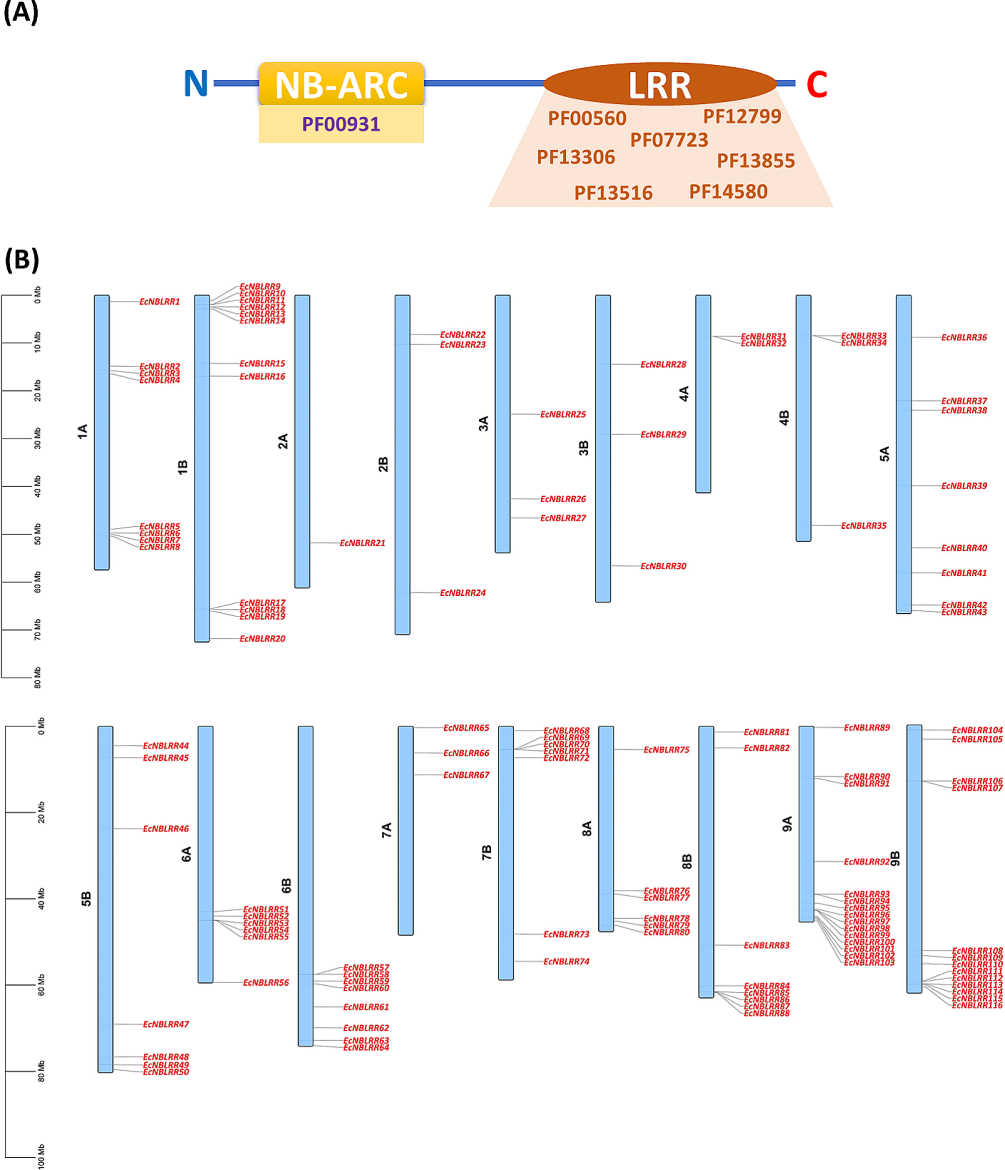
(**A**) The structural features considered for mining NBLRRs in finger millet and related target proteomes. The presence of NB-ARC (PF00931) towards N-terminal and atleast one LRR domain (PF00560, PF12799, PF07723, PF13306, PF13855, PF13516, PF14580) towards C-terminal is considered. (**B**) The distribution of *EcNBLRR* genes in finger millet genome

Genome-wide mining in finger millet resulted in 116 *NBLRR* genes (Additional File 1). Out of 116 EcNBLRRs, 64 are belongs to CNL types (CC-NB-LRR) and five EcNBLRRs showed both CC and RPW8 domain in their N-terminal (RNLs). However, TIR domain not showed any hits in 116 EcNBLRRs. Further, we have mined 159, 241, 198 and 243 NBLRRs in the *Brachypodium distachyon, Oryza sativa, Sorghum bicolor* and *Setaria italica*, respectively (Additional File 2), for undertaking various evolutionary analyses. The physicochemical properties of EcNBLRR proteins showed significant variations in protein length ranging from 354 (EcNBLRR50) to 1452 (EcNBLRR49) amino-acids and molecular weight (MWs) from 40.07 (EcNBLRR50) to 1143.0 (EcNBLRR21) kDa. The maximum and minimum Isoelectric points (*pI*) were recorded as 9.30 (EcNBLRR62) and 4.8 (EcNBLRR29), respectively. The 50.86% of EcNBLRR proteins were acidic (*pI* < 7.0), and 49.14% were found to be basic (*pI* > 7.0). The majority of EcNBLRR proteins were localized in the cytoplasm (50), followed by the nucleus (30), chloroplast (29), whereas lowest EcNBLRR proteins found in the plasma membrane (4), endoplasmic reticulum (2; EcNBLRR3, EcNBLRR23) and peroxisomes (1, EcNBLRR20) (Additional File 3). Further, plotting of *EcNBLRR* genes revealed uneven distribution across the finger millet chromosomes. The maximum number (15) of *EcNBLRR* genes were found on chromosome 9 A (15 genes), followed by 9B (13 genes) and 1B (12 genes). However, only one (*EcNBLRR21*) *EcNBLRR* gene was found on chromosome 2 A (Fig. 1; Additional File 1).

### Structural analysis of ***NBLRR*** genes and proteins in finger millet

**Fig. 2:**
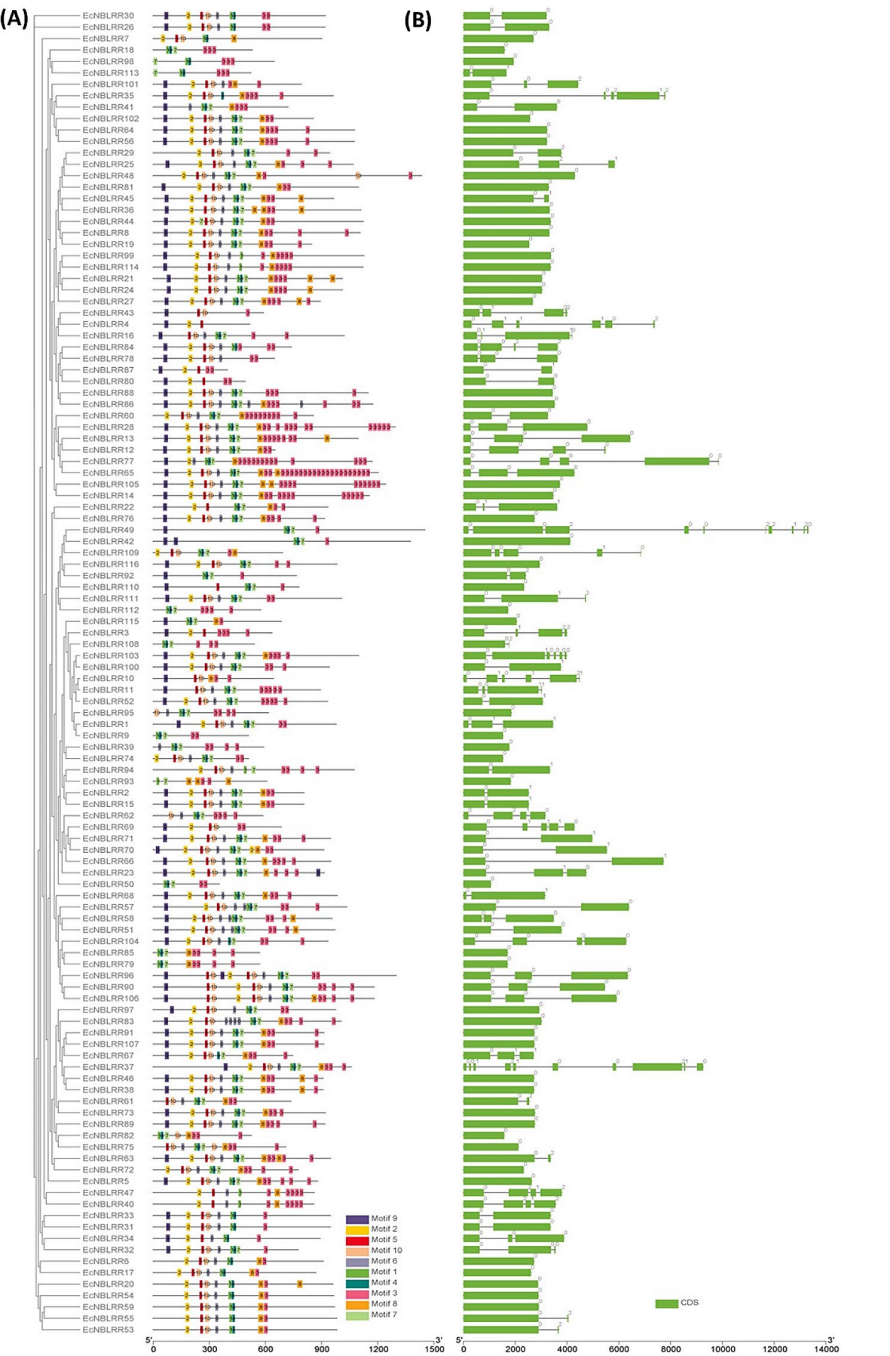
The phylogenetic association, distribution of conserved motifs and gene structure analyses of EcNBLRR proteins and genes. (**A**) Distribution of conserved motifs in EcNBLRR proteins. The best ten conserved motifs in EcNBLRRs are displayed in different colours and numbers boxes. (**B**) *EcNBLRRs* gene structures. The green boxes and the line represent the coding exons and the intron sequences, respectively

The term motif is a conserved amino acid or nucleotide sequence pattern that shows transcriptional or post-translational interactions of proteins or genes with assumed relation to a biological function of the macromolecule. Through the motif identification server MEME, we have fetched the top ten conserved motif structures distributed among 116 EcNBLRR proteins (Additional file 4). Among ten motifs, motifs 3, 1 and 4 were predominantly present in most of the genes, followed by motifs 5, 2, 10, and 7. Motif 8 was present in 72 genes, followed by motif 9, recorded in 83 EcNBLRR. Except for EcNBLRR4 and EcNBLRR7, motif 3 was found in all the EcNBLRR proteins in a repeated arrangement (Fig. 2A).

Structural analysis of *EcNBLRR* genes revealed the exons range from 1 to 10. The *EcNBLRR49* and *EcNBLRR37* showed highest number of exons (*n* = 10), followed by *n* = 6 (*EcNBLRR4*, *EcNBLRR103*, *EcNBLRR10*) and *n* = 5 (*EcNBLRR35*, *EcNBLRR77*, *EcNBLRR109*, *EcNBLRR69*). The lowest number of exons (*N* = 1) was observed in 52 (44.82%), followed by 2 in 28 (24.13%) and 3 in 18 (15.51%) *EcNBLRR* genes. Further, the majority of intronic sequences were found in phase-0 (70.47%), followed by phase-1 (17.71%) and phase-2 (11.81%) (Fig. 2B).

### Evolutionary genetics of NBLRRs in finger millet and related Gramineae members

#### Phylogenetic analysis of NBLRRs

**Fig. 3:**
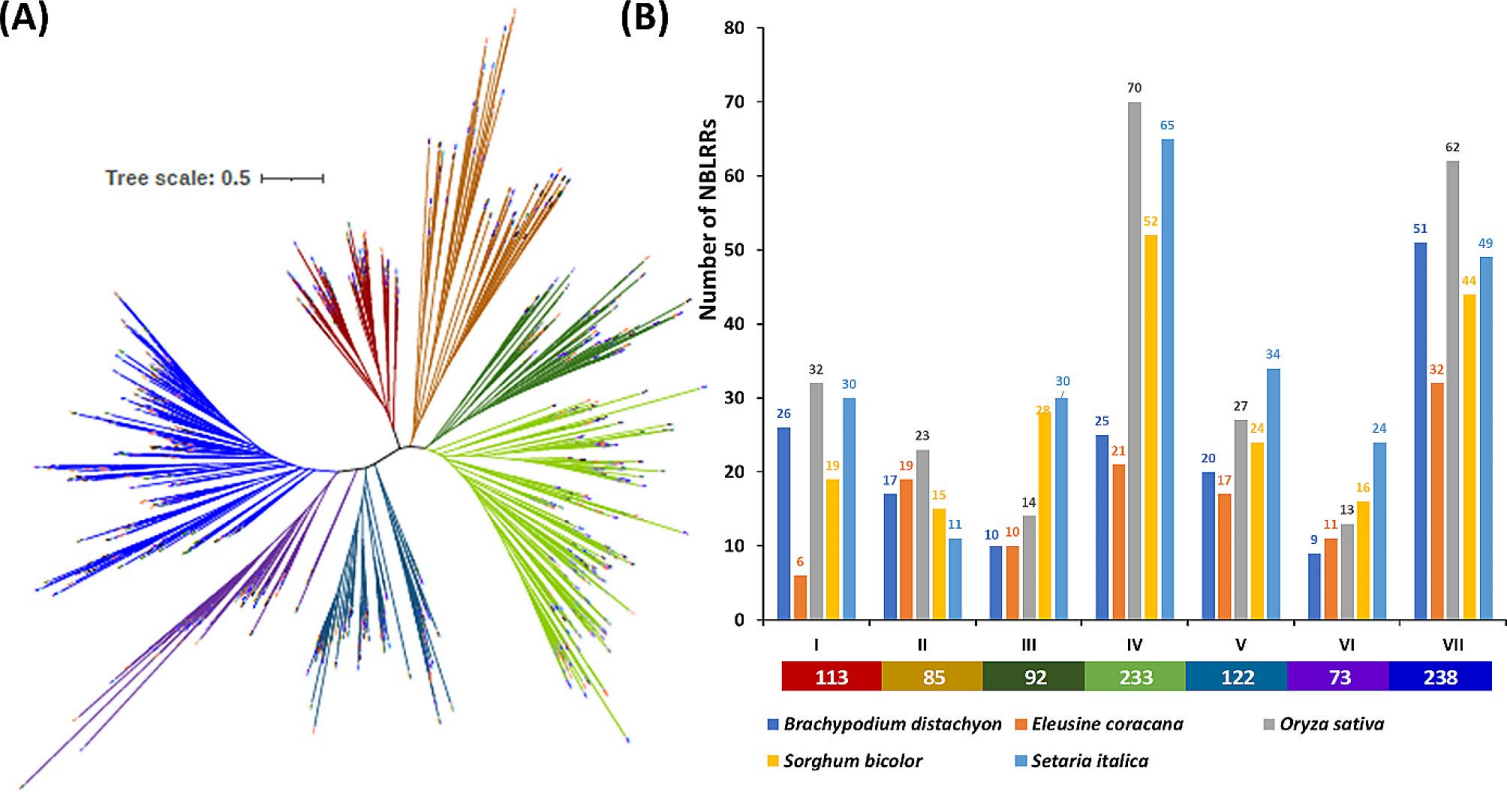
(**A**). The unrooted phylogenetic tree of 957 NBLRRs from four crop species, *viz*., finger millet, rice, sorghum, foxtail millet and a model grass, purple false brome. The clusters I, II, III, IV, V, VI and VII are represented by dark red, dark orange, dark green, light green, loyal blue, purple and blue colours, respectively. Phylogenetic trees were constructed using IQTREE.v.16.12 using the Maximum Likelihood method with 1000 bootstrap replicates (Note: For a high-resolution phylogenetic tree, please refer to supplementary information, Additional File 5). (**B**). The cluster-wise distribution of 957 NBLRRs among the five target species. The values below the cluster letters indicate the total number of NBLLRs in the cluster

The VT + F + R10 model was selected based on Akaike and Bayesian information criteria to perform the phylogenetic analysis of NBLRR protein sequences. Phylogenetic analysis showed the clustering of 957 NBLRR proteins from five Gramineae members into seven clusters, i.e. I to VII (Fig. 3; Additional File 5). Further, the NBLRRs showed uneven distribution across the different clusters. Cluster-VII grouped the highest *NBLRRs* (*n* = 238), followed by cluster-IV with 233 *NBLRRs*. Whereas the cluster-VI emerged as a smaller cluster with 73 NBLLRs. The clusters V, I, III and II showed 122, 113, 92 and 85 NBLRRs, respectively. The overall topology of the phylogenetic tree showed the mixed grouping pattern with representation of NBLLRs from all the species in each cluster (Fig. 3). Thus, the absence of species-specific grouping and the presence of mixed grouping of NBLRRs of the target species suggest their evolution in the ancestral lineages of the target species.

#### Duplication of ***NBLRRs*** in finger millet

**Fig. 4:**
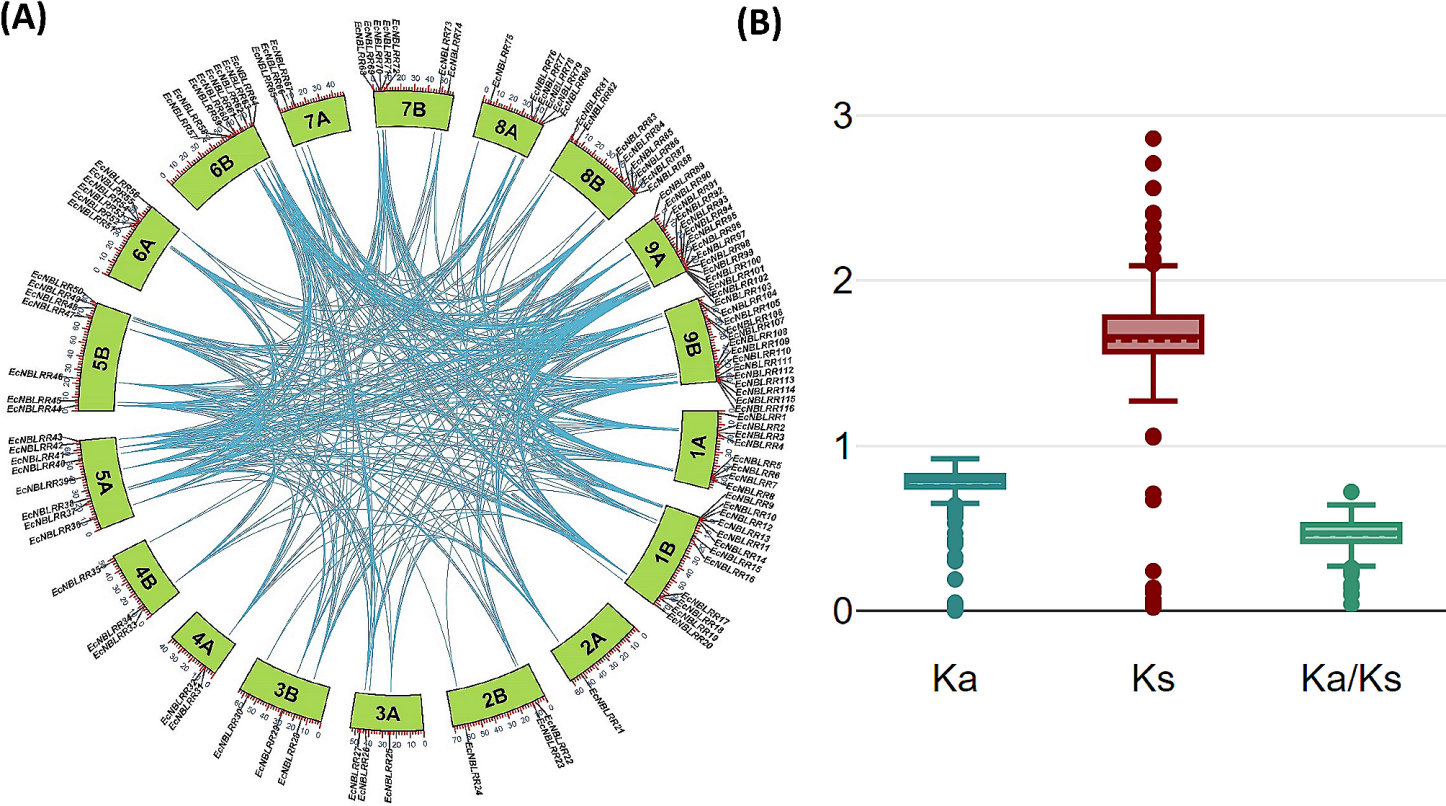
(**A**) Schematic representations of the chromosomal distribution and duplication relationships of NBLRR genes in the finger millet genome (**B**) The box and whisker plots for non-synonymous substitutions (Ka), synonymous substitutions (Ks) and the ratio of non-synonymous and synonymous substitutions (Ka/Ks) among the *NBLRR* paralogs of finger millet

The duplication analysis was performed to study the divergence of *NBLRR* genes in the finger millet genome. The results revealed 473 duplication pairs among the 116 *EcNBLRRs* distributed across the 18 chromosomes. Further, the results showed that whole-genome duplication (WGD) is the primary evolutionary force in the divergence of *EcNBLRRs*. (Fig. 4A). Further,52.64% of paralog pairs showed loss of CC or CC_R_ domain in one of the counterparts, whereas 47.36% showed similar kinds of NBLRR structures (Additional File 6). The mean synonymous substitutions (Ks = 1.64) were found predominately in *EcNBLRR* paralogs over the mean non-synonymous (0.75) substitutions, suggesting the prominent role of purifying selection (Ka/Ks = 0.46) on the divergence of *NBLRRs* in finger millet genome (Fig. 4B; Additional File 6).

#### Orthologs, synteny and divergence of NBLRRs among the five target species of Gramineae

**Fig. 5:**
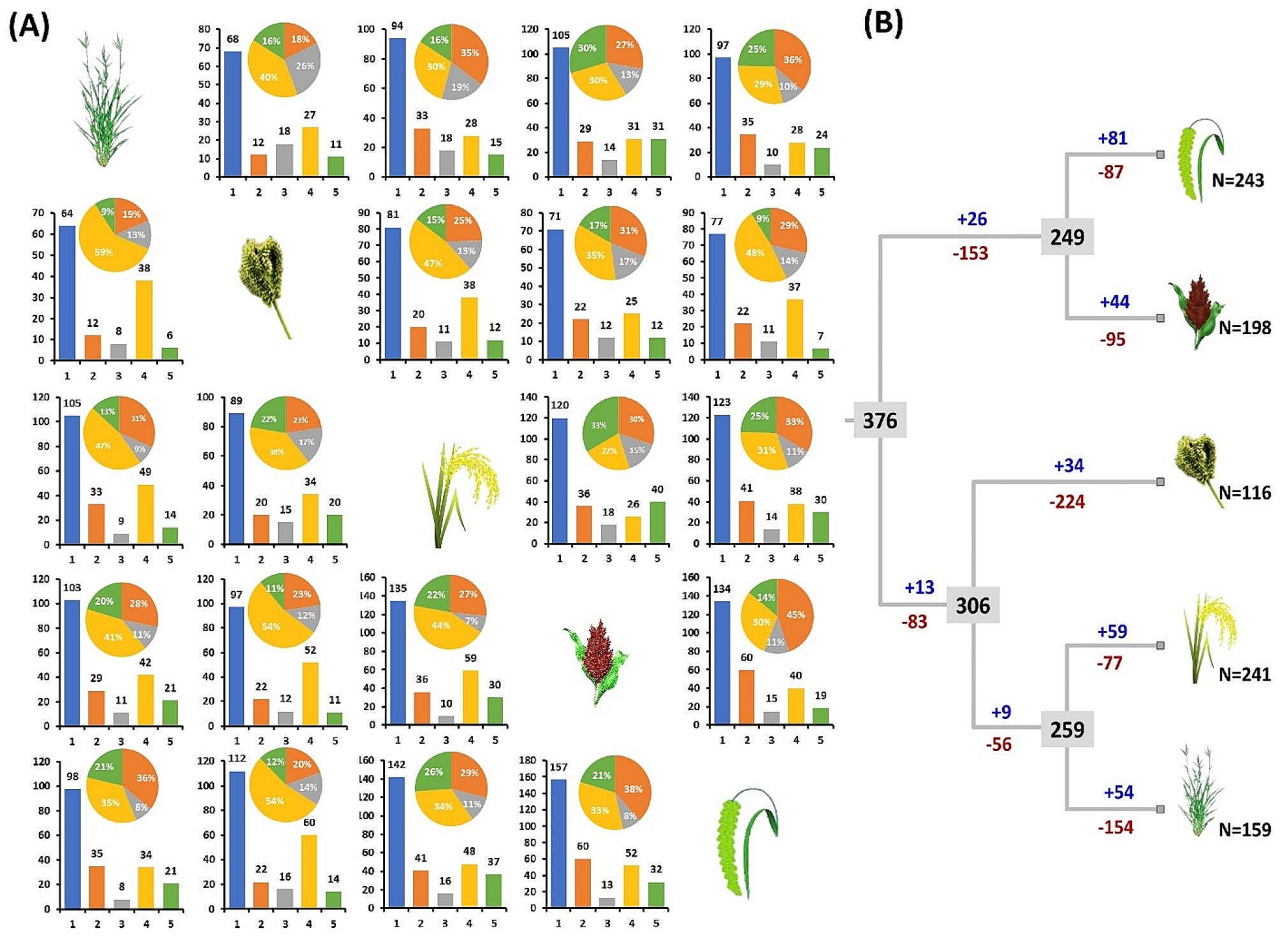
The orthology of NBLRRs among finger millet, rice, sorghum, foxtail millet and a model grass purple false brome. (**A**) The bar graph shows the total and various kinds of orthologs observed among the five target species. The Pie diagram shows the percentage of various kinds of orthologs in each species pair. The blue bars represent the total number of orthologs. The orange, grey, yellow and green bars and slices of Pie diagrams represent the one-to-one, one-to-many, many-to-one and many-to-many orthologs. (**B**) The schematic representation of gain and loss of *NBLRRs* in five target species of Gramineae. The numbers in the rectangles of phylogenetic nodes represent the number of *NBLRRs* in the ancestors, and the blue and red numerals with plus (+) and minus (–) sign correspondingly represent the gain and loss of NBLRR genes, respectively

Homology-based search by OrthoFinder identified 1761 *NBLRR* ortholog pairs among finger millet, rice, sorghum, foxtail millet and a model grass purple false brome. The maximum number of *NBLRR* orthologs was identified between the foxtail millet and sorghum (157), followed by sorghum and rice (135) and sorghum and foxtail millet (134). Further, the *EcNBLRRs* showed maximum orthology with rice (81), followed by foxtail millet (77), sorghum (71) and purple false brome (64) (Fig. 5A**).**

The extent of co-orthology, or the degree of duplications/loss reported with relationship cardinality among the pairwise combinations of orthologs. The one-to-one pairwise orthology means both the genes of an ortholog pair in the pair have only one copy in the other corresponding species. The gene of interest in one species has more than one ortholog in the other species, resulting in one-to-many orthology owing to gene duplications or gains in an ancestor of the other species. In contrast, the vice versa condition (many-to-one orthology) indicates the loss of genes in the ancestral lineage of the other species. Furthermore, the many-to-many orthology occurs owing to lineage-specific duplications in both species. The *EcNBLRRs* showed the highest percentage of many-to-one orthologs (35–59%) with all other target species. Similarly, many-to-one orthologs predominated among NBLLR orthologs, except the predominance of one-to-one orthologs for *BdNBLLRs-OsNBLRRs* (35%), *BdNBLLRs-SiNBLRRs* (36%), *OsNBLLRs-SiNBLRRs* (33%), *SbNBLLRs-SiNBLRRs* (45%), *SiNBLLRs-BdNBLRRs* (36% and SiNBLLRs-SbNBLRRs (38%), and many-to-many orthologs for *OsNBLRRs-SbNBLRRs* (33%) and *BdNBLLRs-OsNBLRRs* (30%). Interestingly, 18 of 20 (90%) ortholog combinations showed the lowest one-to-many orthologs (7–17%) except *BdNBLLRs-EcNBLRRs* (26%) and *BdNBLLRs-OsNBLRRs* (19%) (Fig. 5A). Furthermore, to validate the outcome of the orthologs analysis, we have reconciled the target species tree with the NBLRRs tree. The results showed the maximum loss (307) of NBLRRs in the finger millet lineage compared to the rest of the species, which is in accordance with the high percentage of many-to-one orthologs. Furthermore, the divergence of NBLRRs showed the predomination of gene losses over gene gain events owing to the high proportion of many-to-one orthologs and the lowest representation of one-to-many orthologs. Thus, the loss of gene events diverged the NBLRRs in the lineage of target Gramineae species (Fig. 5B).

**Fig. 6:**
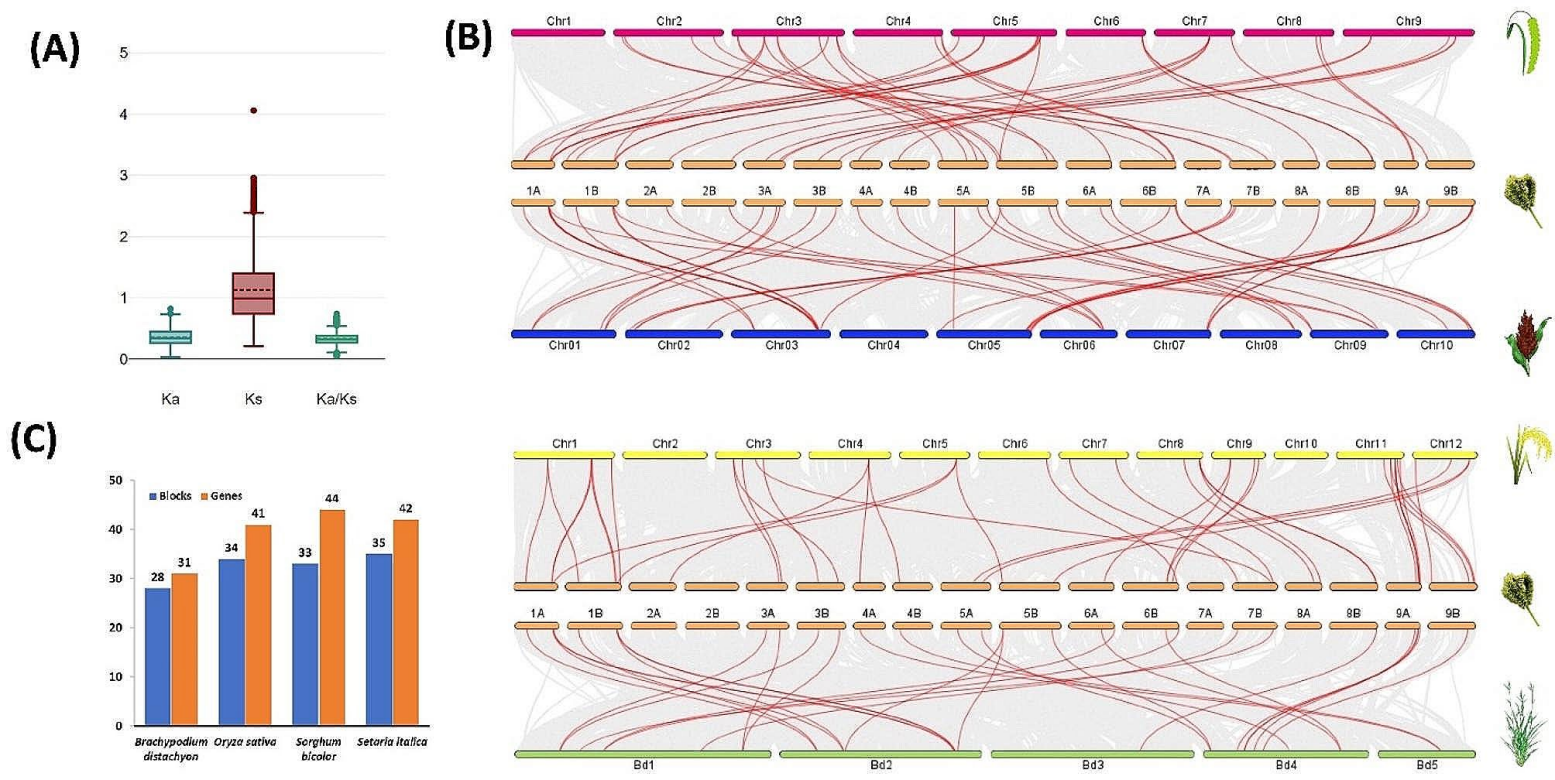
(**A**) The box and whisker plots for non-synonymous substitutions (Ka), synonymous substitutions (Ks) and the ratio of non-synonymous and synonymous substitutions (Ka/Ks) among the NBLRR ortholog of target species. (**B**) The collinearity relationships between *NBLRR* genes of finger millet with other target species. The orange, pink, blue, yellow and green horizontal bars represent the chromosomes of finger millet, foxtail millet, sorghum, rice and a model grass purple false brome, respectively. (**C**) The bar plots show the syntenic summary of NBLRRs between finger millet and other target species

The NBLLR ortholgs showed an average substitution of 31.99% with a range of 6.15 (*SbNBLRR45-SiNBLRR243*) to 77.09% (*OsNBLRR88-SbNBLRR188*). The Ka/Ks ratio was obtained with a statistical significance of *P* > 0.001 for *NBLRR* ortholog pairs among the five target species. Selection pressure analyses calculated with the GY-HKY substitution model showed a mean Ka/Ks ratio of 0.33 and a range of 0.04 (*OsNBLRR241-SbNBLRR158*) and 0.74 (*EcNBLRR9-SiNBLRR200*). Thus, the NBLRR orthologs evolved under strong purifying selection (Ka/Ks < 1.0) (Fig. 6A; Additional File 7).

To dissect the syntenic association of *NBLRR* genes among the target genomes, the syntenic and collinear analysis was performed. The *EcNBLRRs* showed the lowest syntenic association with *B. distachyon*, (28 blocks with 31 gene pairs) compared to *O. sativa* (34 blocks with 41 gene pairs), *S. bicolor* (33 blocks with 44 gene pairs) and *S. italica* (35 blocks with 42 gene pairs) (Fig. 6B and C; Additional File 8).

### Functional analysis of NBLRRs in finger millet

#### The ***cis***-acting elements analysis of NBLRR promoter sequences in finger millet

**Fig. 7:**
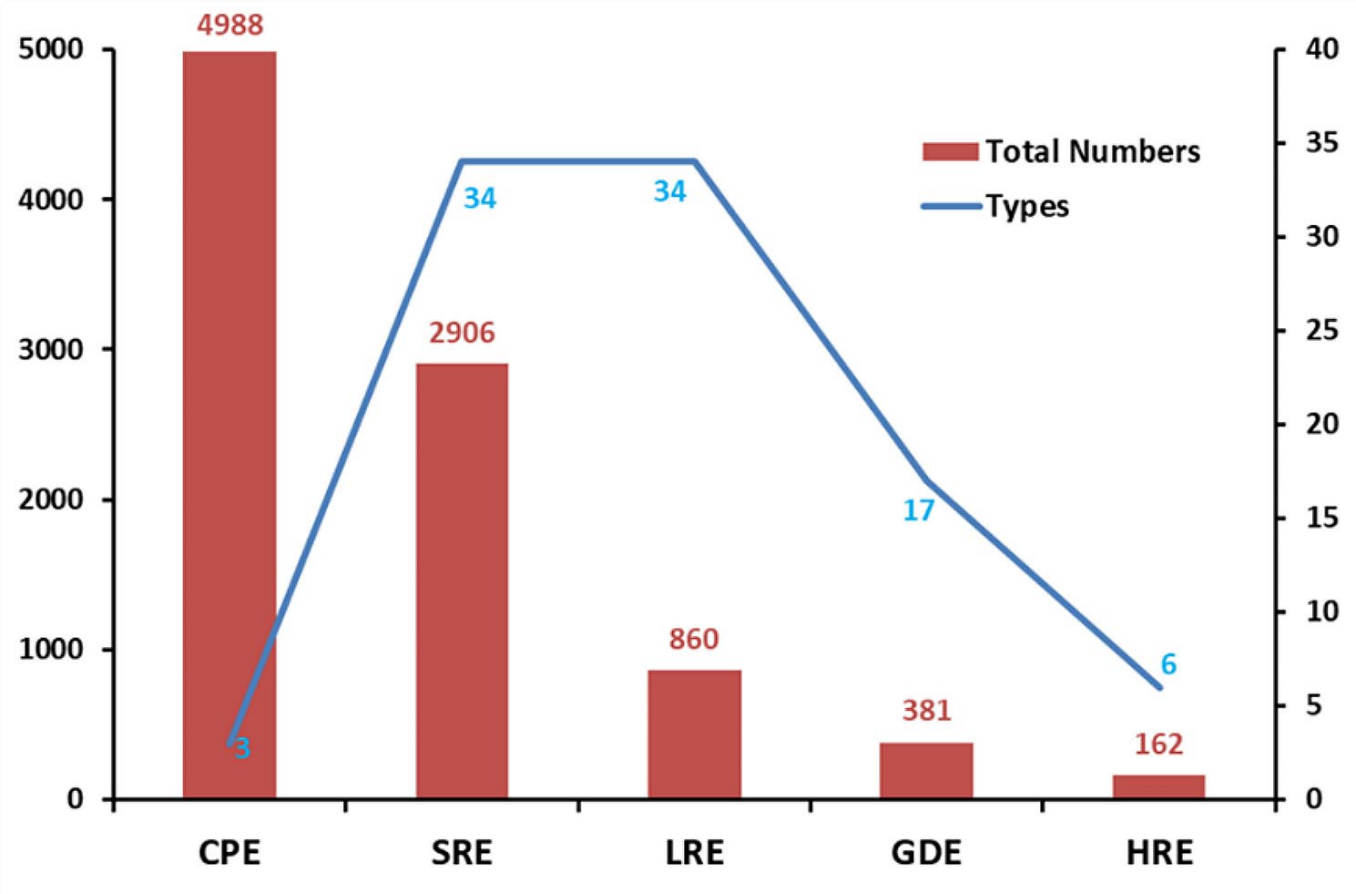
The different kinds and numbers of cis-acting elements identified in the 1.5 kb upstream promoter sequences of *EcNBLRRs*. Note: CPE-core promoter cis-acting elements, SRE- stress responsive cis-acting elements, LRE- light responsive cis-acting elements, GDE- growth and development related cis-acting elements and HRE- hormone responsive cis-acting elements

The *cis*-acting elements predicted in the 1.5 kb upstream promoter sequences of *EcNBLRRs* were grouped into five different functional categories such as (i) core promoter, (ii) growth and development, (iii) hormonal-responsive, (iv) light-responsive and (v) stress-responsive elements (Fig. 7). The results revealed that all the promoter sequences of *EcNBLRR* genes displayed a higher occurrence in core elements such as *CAAT-box* (*N* = 2571), followed by *TATA-box* (*N* = 2186) and *AT ~ TATA-box* (*N* = 231), respectively (Additional File 9). Among the growth- and development-related elements, *CAT*-box found in higher occurrence (*N* = 75) known to regulate meristem expression, followed by *O*_*2*_-site (*N* = 56) related to zein metabolism regulation, and circadian element control the circadian cycle, *GCN4*-motif regulate the endosperm growth and the element *RY* implicated in seed-specific regulation (Additional File 10).

Further, 162 copies of 6 hormonal-responsive *cis*-acting elements were categorized for auxin (*AuxRR*-core, *TGA*-element, *TGA*-box) and gibberellins (*GARE*-motif, *TATC*-box, *P-box*) responsiveness, respectively. Among the hormonal-responsive *cis*-acting elements, the gibberellins regulating elements were found to be more (*N* = 91), followed by auxin (*N* = 71) (Additional File 11). A total of 860 light-responsive *cis*-acting elements falling under 34 types were identified. The maximum number of light-responsive elements identified are *G-box* (*N* = 279), followed by *Box-4* (*N* = 90), *GT1-motif* (*N* = 68), *TCT-motif* (*N* = 62) and *GATA*-motif (*N* = 56) (Additional File 12). The 34 stress-responsive *cis*-acting elements were present in 2,906 copies across the *EcNBLRRs* promoter sequences. The abscisic acid-responsive *ABRE* (*N* = 251) elements were more predominant stress-responsive elements, followed by *CGTCA-motif* (*N* = 210) related to exogenous methyl jasmonate (MeJA) responsive element and anaerobic responsive *ARE* (*N* = 154) elements. Further, the *TCA* (*N* = 39) and *SARE* (*N* = 2) related to salicylic acid (N = SA) responsiveness were reported. Additionally, *TC-rich repeats* (*N* = 44), *MBSI* (*N* = 6) and *WUN-motif* (*N* = 3) elements related to defence and stress-responsive, flavonoid gene synthesis and wound-responsiveness, respectively, were distributed among the promoter sequences of *EcNBLRRs* (Additional File 13).

### Expression analysis of ***EcNBLRR*genes against*M. grisea*infection**

**Fig. 8:**
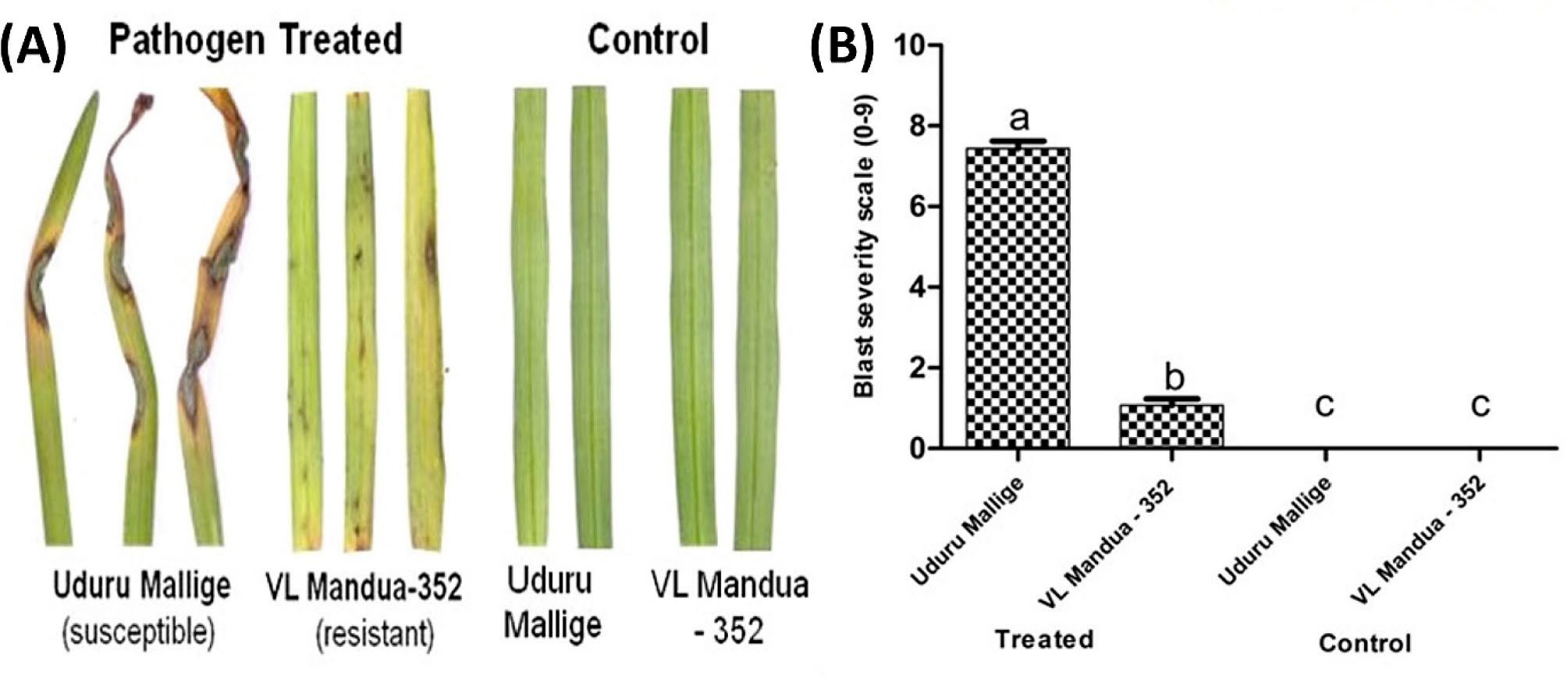
(**A**) The phenotypic response of finger millet cultivars Uduru Mallige and VL Mandua-352 showing susceptible and resistant disease reactions, (**B**) The bar graph plotted using severity of blast lesion as per 0–9 scale grade in the contrasting finger millet cultivars Uduru Mallige and VL Mandua-352

Upon *M. grisea* inoculation, the minute brown spots appeared in susceptible genotype Uduru Mallige at 3–4 days post inoculation (dpi) and thereafter, enlarged to develop a typical spindle-shaped blast lesion with grey or white centre characteristics at 7–8 dpi. Subsequently, these lesions spread to the entire leaf lamina and become dead leaves for which disease scale values 8 and 9 were assigned. Conversely, the resistant genotype VL Mandua-352 developed a hypersensitive reaction (HR) with minute brown or little elongated spots randomly scattered at 4–6 dpi. However, these spots failed to expand further, for which scales 2 and 3 were assigned (Additional File 14). The percent disease incidence (PDI) of blast severity revealed 82.59% and 11.85% of blast incidence in Uduru Mallige (susceptible) and VL Mandua-352 (resistant), respectively. Control plants treated with sterile water had no symptoms (Fig. 8; Table 1).

**Table 1:** Mean phenotypic response of susceptible (Uduru Mallige) and resistant (VL Mandua-352) cultivars of finger millet to infection of *M. grisea* isolate Ragi Almora (FMg_Al). Note: The data was recorded on thirty replications. CD – critical difference; SE(d) – standard error of deviation; SE(m) – standard error of the mean

Finger millet cultivars		Percent disease incidence (PDI)^*^
1) cv. Uduru Mallige - Pathogen inoculated		82.59^a^
2) cv. Uduru Mallige – Water inoculated (control)		0.00^c^
3) cv. VL Mandua − 352 - Pathogen inoculated		11.85^b^
4) cv. VL Mandua − 352 - Water inoculated (control)		0.00^c^
***CD (P = 0.05)***		**0.34**
***SE(d)***		**3.17**
***SE(m)***		**0.29**

**Fig. 9:**
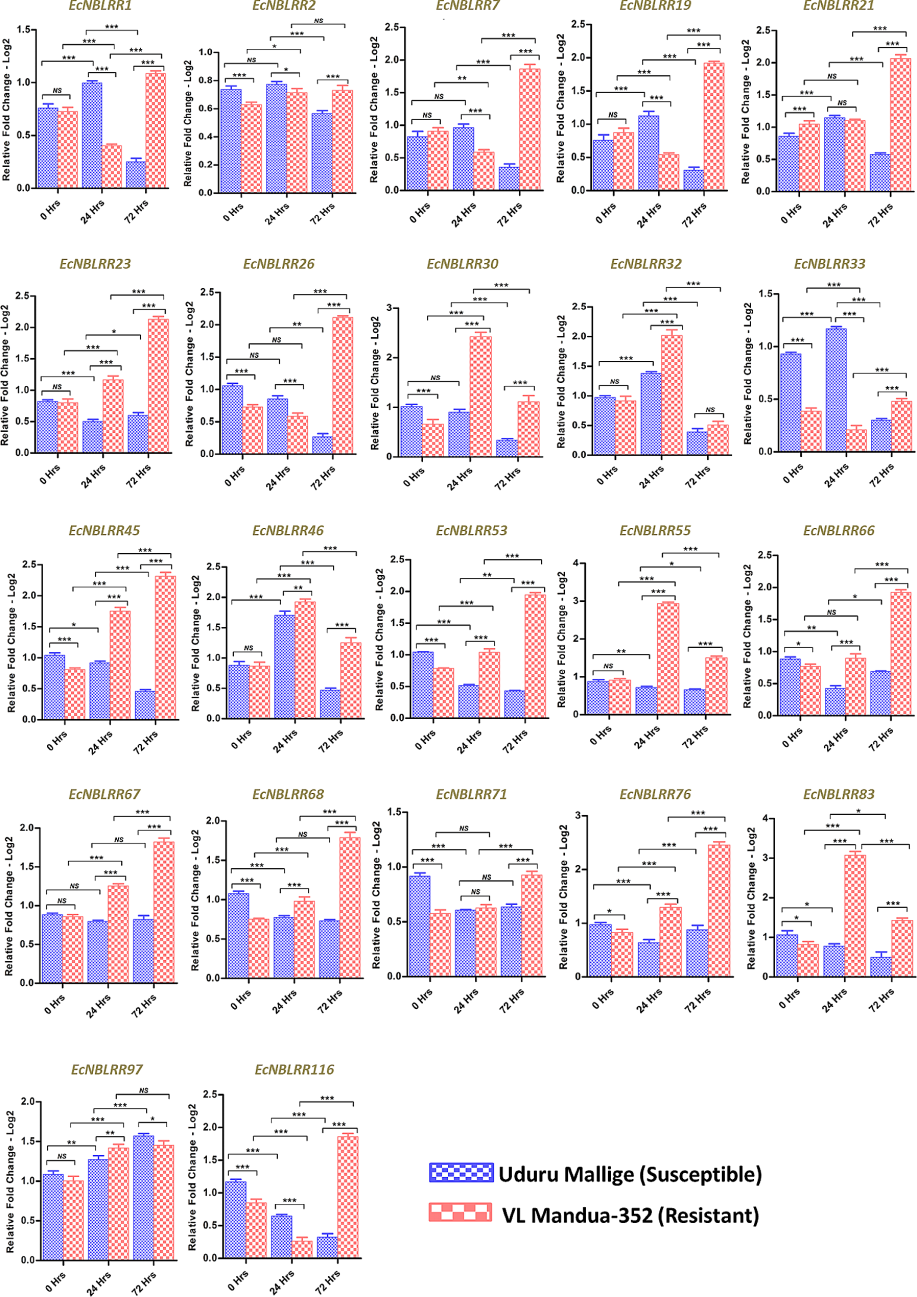
The relative expression level of 22 *EcNBLRR* genes in the Uduru Mallige and VL Mandua-352 genotype showing contrasting results in response to *M. grisea* isolate Ragi Almora (FMg_Al) infection at 24 and 72 hpi during RT-qPCR analysis. The error bars represent the deviations from three biological replicates; the asterisk indicated significant differences at *P* < 0.01 (*), *P* < 0.001 (**) and *P* < 0.0001 (***); NS - no significant difference and hpi -hours post-inoculation

The *EcNBLRRs* expression analysis was conducted to decipher the functional response of *EcNBLRRs* to blast in the genotypes Uduru Mallige (susceptible) and VL Mandua-352 (resistant) showing contrasting disease reactions (Fig. 8). A total of eight *EcNBLRR* genes showed significantly enhanced expression with the time of infection (*EcNBLRR2, EcNBLRR23, EcNBLRR26, EcNBLRR45, EcNBLRR53, EcNBLRR67, EcNBLRR68 and EcNBLRR76*) in resistant genotype VL Mandua-352. *EcNBLRR21, EcNBLRR66* and *EcNBLRR71* showed significant expression at 72 hpi; however, no significant difference was observed between 0 and 24 hpi. Whereas, in the case of *EcNBLRR97*, the fold change between 0 and 24 hpi was significant, while at 24 and 72 hpi remained unchanged. Also, *EcNBLRR97* showed enhanced expression in both Uduru Mallige (susceptible) and VL Mandua-352 genotypes. The susceptible genotype Uduru Mallige has shown a significant inverse relationship between the time point of inoculation and expression of nine *EcNBLRRs* (*EcNBLRR23, EcNBLRR26, EcNBLRR45, EcNBLRR53, EcNBLRR55, EcNBLRR71, EcNBLRR76, EcNBLRR83*, and *EcNBLRR116*). Similarly, non-significant expression was observed between 24 and 72 hpi in the case of *EcNBLRR68* and 0 and 24 hpi for *EcNBLRR30.* On the contrary, the *EcNBLRR67* showed no significant expression in Uduru Mallige across the time points (0–72 hpi) (Fig. 9).

The genes *EcNBLRR30, EcNBLRR32, EcNBLRR46, EcNBLRR55* and *EcNBLRR83* in VL Mandua-352 showed highest significant expression at 24 hpi than 72 hpi, whereas the vice-versa for *EcNBLRR1, EcNBLRR7, EcNBLRR19, EcNBLRR21, EcNBLRR23, EcNBLRR26, EcNBLRR33, EcNBLRR45, EcNBLRR53, EcNBLRR66 EcNBLRR67, EcNBLRR68, EcNBLRR76* and *EcNBLRR116* in same genotype. Furthermore, the susceptible Uduru Mallige showed peak significant expressions of *EcNBLRR1, EcNBLRR19, EcNBLRR21, EcNBLRR32, EcNBLRR33*, and *EcNBLRR46* at 24 hpi than 72 hpi, whereas *vice-versa* pattern for *EcNBLRR97* (Fig. 9).

The *EcNBLRR83* gene in VL Mandua-352 showed 3.07 FC expression compared to 0.77 FC in susceptible genotype Uduru Mallige at 24 hpi (*P* = 0.0001). The *EcNBLRR55* recorded higher FC (2.94) in VL Mandua-352 against 0.71 FC in susceptible genotype Uduru Mallige induced at 24 hpi (*P* = 0.0001). Similar patterns were followed by *EcNBLRR30* (FC: 2.42) and *EcNBLRR32* (FC: 2.42) in VL Mandua-352 compared with Uduru Mallige (*EcNBLRR30*: 0.9; *EcNBLRR32*: 1.38) at FC in 24 hpi (*P* = 0.001). However, all the above genes showed significantly decreased FC expression at 72 hpi (*P* = 0.001). The *EcNBLRR76* showed 2.45 fold expression in VL Mandua-352 compared to 0.87 FC in susceptible cultivar Uduru Mallige at 72 hpi (*P* = 0.0001). Similar trends of expressions were also followed by *EcNBLRR45* (FC: 2.31), *EcNBLRR26* (FC: 2.13), *EcNBLRR23* (2.11), and *EcNBLRR21* (2.06), recorded higher fold expression in VL Mandua-352 compared to FC expressions in susceptible line Uduru Mallige (*EcNBLRR45*: 0.46; *EcNBLRR26*: 0.60; *EcNBLRR23*: 0.26 and *EcNBLRR21*: 0.57) (*P* > 0.0001) (Fig. 9). The statistically significant (*P* > 0.001) and more than the 2-fold expression of *EcNBLRR30, EcNBLRR32, EcNBLRR55* and *EcNBLRR83* were observed at 24 hpi and *EcNBLRR21, EcNBLRR23, EcNBLRR26, EcNBLRR45* and *EcNBLRR76* at 72 hpi compared to control as well as the expression levels in susceptible genotype Uduru Mallige indicating these could be putative target genes associated with blast resistance in finger millet (Fig. 9). Further, the six of nine candidate EcNBLRRs, *viz.,* *EcNBLRR21*, *EcNBLRR26*, *EcNBLRR30, EcNBLRR45, EcNBLRR55* and *EcNBLRR76* showed CC-NB-LRR structures. Whereas* EcNBLRR23*, *EcNBLRR32* and *EcNBLRR83* showed NB and LRR domains.

## Discussion

Finger millet is one of the most important millets, but the production is constrained by blast infection, leading to severe yield losses. Thus, a better understanding of host-pathogen interactions is crucial in the genetic improvement of blast resistance in finger millet. Several genomic regions carrying resistance (*R*) genes against potentially important pathogens have been reported in several plant species [32]. Among various *R* genes, the *NBLRR* genes are the immune receptors that recognize the pathogen effector molecules (specific/non-specific) and trigger defence responses in host plants [33]. Studies on *NBLRRs* have already been extensively explored on various plant species, including *Arabdiopsis thaliana* [17], *Oryza. sativa* [14], *Zea mays* [34], *Populus trichocarpa* [15], *Vitis vinifera* [13], *Dioscorea rotundata* [26], Chinese cabbage [27], *Cucumis sativus* [35] etc. in response to various disease resistance. An effort to capture the *NBLRRs* in finger millet using only NB-ARC domain-specific degenerative primers using PCR resulted in 57 NBSLRRs [30]. However, identifying the *NBLRRs* on a genome-wide scale and detailed characterization and expression in response to *M. grisea* infection in finger millet has not yet been explored. Hence, the current investigation aimed to characterize and validate the *NBLRR* genes against *M. grisea* infection in finger millet.

Here, we identified 116 non-redundant NBS-encoding *R* genes with at least one leucine-rich repeat domain toward the carboxy-terminal end in the finger millet genome (Additional File 1) using both homology-based blast search and PFAM-based HMM-scan and HMM-search. In the current investigation, the EcNBLRRs not showed any TIR domains, on other hand ~60% of EcNBLRRs showed N-terminal located CC domain. The previous studies showed absence of TIR domain in the NBLRRs of cereal species indicating the loss of TNLs in cereal linages from the early angiosperm ancestors [36, 37]. The CC-NBLRRs play a major role in assigning resistance to various fungal pathogen in crops. For instance, *Lr10* [38] for leaf rust. *Yr10* [39] and *RPM1* [40] for stripe rust and *Pm21* for powdery mildew resistances [41] in wheat, and *Pb1* [42] for panicle blast resiatnce in rice. The NBLRRs with a RPW8-like CC domain are referred to as CCR-NBLRRs or RNLs [43, 44]. The five EcNBLRRs, *viz.,* EcNBLRR12, EcNBLRR65, EcNBLRR77, EcNBLRR102 and EcNBLRR105 showed RPW8 domins with CC structures at N-Terminal. The over expression of RNLs (*MdRNL1* to *MdRNL5*) in Apple showed enhanced resistance to *Alternaria* leaf spot, whereas their silencing in the resistant cultivars showed increased disease incidence [45].

The physicochemical properties of EcNBLRR proteins showed high variation in protein length (354–1452 aa) and molecular weights (40.07–1143.00 kDa). The structural variation of EcNBLRRs could be associated with their functional diversification in dealing with various finger millet pathogens. The proper subcellular localization or targeting of R proteins is a prerequisite for assigning resistance to pathogens. The maximum EcNBLRRs showed subcellular localizations in the cytoplasm (50) and nucleus (30) (Additional File 3). Many reports showed the localization of the R proteins in the nucleus and cytoplasm under pathogen infection, even without clear nuclear localization signals (NLS) in most of the R protein sequences [46, 47]. However, in some of the cases, the presence of NLS is shown to be essential for resistance reactions in barley, tobacco and Arabidopsis R proteins [46, 48–50]. Few studies showed differentiated immune responses from cell death signalling. For instance, the suppressed cell death and enhanced immune response activity of R protein MLA10 results when targeted to the nucleus, but cell death is enhanced when it is in the cytoplasm [51]. Further, the structural analysis of *EcNBLRRs* variation in the number of exons per EcNBLRR (1 to 10) and intronic phases. Most introns were found at phase-0 (70.47%), followed by phase-I (17.71%). The phase-0 intron does not disrupt a codon, whereas the phase-1 and phase-2 intron disrupt a codon between the first and second, and second and third bases, respectively [52]. The distribution of intron phases is non-uniform: phase-0 introns occur most frequently, and phase-2 introns occur least frequently, suggesting that the proportion of phase-0 introns increased during evolution [52–55].

To study the evolutionary patterns of NBLRRs among five target species with the same approach, we have mined 159, 241, 198 and 243 orthologs of *B. distachyon, O. sativa, S. bicolor* and *S. italica*, respectively. However, the mined NBLRRs homologs were part of previous reports in *O. sativa* (480 [14]) and *B. distachyon* (126, [56]), and the discrepancies in the number could be due to mining approaches employed, the e-values in BLAST search, HMM-search and HMM-scan, and updated genome assemblies. The NBLRRs evolve rapidly to cope with the dynamic pathogen populations for plant survival [57], Thus, strong natural selection facilitates R-genes’ divergence through gene gain and loss events [58, 59]. The topology of the NBLRR phylogenetic tree mostly showed mixed clustering patterns of NBLRRs, suggesting their evolution in the ancestral lineages of the target species of Gramineae. The NBLRRs could share conserved homologous sequences across the Gramineae species much before the evolutionary divergence of the grasses [30]. The subsequent divergence of NBLRRs occurred through evolutionary forces. Further, supporting evidence was observed through the predominance (~40%) of many-to-one orthologs among the NBLRR orthology and prominent NBLRR losses in the evolutionary lineage of target species in the gene loss and gain analysis (Fig. 6). Similarly the high frequency of gene loss events in diversification of *NBLRRs* are observed in other members of Poaceae, *viz.,* rye (-262), barley (-360) and *Triticum urartu* (-230) [60] Gene loss is one of the prominent sources of genetic variation in plants [61]. Thus, predominantly lineage-specific gene losses followed by gene gains shaped the divergence of *NBLRRs* in plants [14].

The whole genome duplications (WGDs) expanded *EcNBLRRs* under strong purifying selection in the finger millet. The WGD, followed by functional diversification, played a prominent role in the expansion, evolution, and diversification of various polyploid gene families [62]. There are reports on both rapid and slow evolutionary patterns of NBLRRs in plants with frequent and rare gene conversions, respectively [63, 64]. This indicates that gene duplication and unequal crossing-over are followed by density-dependent purifying selection in the evolution of *NBLRRs* [64]. Thus, the functional diversification of duplicated *NBLRRs* supports adaptation and has likely been favoured by natural selection during evolution. Synteny gives the genomic framework for conserved homologs and their order between the different species’ genomes. The low syntenic association of *EcNBLRRs* with the NBLRRs of model plant species, *B. distachyon*, compared to *O. sativa, S. bicolor* and *S. italica* mostly followed the target species lineages (Figs. 5B and 6B) owing to close evolutionary association. The maximum shared synteny between finger millet and genomic fragments from *O. sativa, S. bicolor* and *S. italica* species is originated from a more immediate identical ancestor than finger millet and *B. distachyon* in evolutionary lineage [65].

The scanning of *EcNBLRR* promoter sequences showed significant occurrences of stress-, hormonal- and light-responsive *cis*-elements besides core and growth & development related *cis*-elements (Fig. 6). The results hint at the additional direct and indirect association of *EcNBLRRs* with various developmental and stress-responsive activities in addition to coding for disease resistance. The stress-responsive phytohormones, *viz.,* salicylic acid (SA), jasmonic acid (JA), and abscisic acid (ABA), mediate the signal transduction pathways for defences against diseases and other stresses in plants [66]. For instance, enhanced expression of an *NBLRR* gene *ADR1* in the presence of salicylic acid resulted in drought tolerance [67]. Supportingly, 39 and 2 *EcNBLRRs* showed salicylic acid-responsive *SARE* and *TCA* elements, respectively. Similarly, salicylic acid-responsive TCA-element between − 563 bp and − 249 bp upstream of an R gene *OsPIANK1* regulates resistance mechanism to *M. oryzae* infection [68]. The *ABRE* elements were present in higher proportion among the *EcNBLRRs* promoters (291), which acts as a binding site for ABA-dependent transcription factors to regulate various stresses [69, 70]. The *G-box* is a ubiquitous light regulatory element in many of the promoters [71] and is highly represented light-responsive *cis*-element (279) among the *EcNBLRRs* promoters, followed by *Box-4* (90), *GT-1* (62) motif, etc. Several studies have shown the essential roles of a particular spectrum of light in promoting plant defence against various pathogen infections [72]. For instance, the red-light spectrum enhances the plants’ resistance to various pests and pathogens [73]; however, the detailed molecular basis still needs to be elucidated. Recently, several researchers showed *AS-1, G-box* and *W-box* as pathogen-inducible *cis*-regulatory elements in the promoter regions of* R*-genes [74–77]. Thus, the diverse *cis*-acting elements could modulate the expression of *EcNBLRRs* towards direct and indirect regulation of disease resistance in finger millet.

The qRT-PCR expression analysis of 22 *EcNBLRRs* in response to *M. grisea* infection showed typical genotypic- and infection-specific expression patterns in finger millet. The nine *EcNBLRR* genes were showed enhanced fold expressions at 24 hpi (*EcNBLRR83*: 3.07; *EcNBLRR55*: 2.94; *EcNBLRR30*: 2.42; *EcNBLRR32*: 2.02) and 72 hpi (*EcNBLRR76*: 2.45; *EcNBLRR45*: 2.31; *EcNBLRR26*: 2.13; *EcNBLRR23*: 2.11; *EcNBLRR21*: 2.06) in the resistant genotype VL Mandua-352 compared to susceptible genotype Uduru Mallige. The compatible interactions between the susceptible host and the pathogen result in a substantial increase in the fungal growth on the host. On the other hand, incompatible interactions between resistant genotype and pathogen result in resistance reaction owing to the arresting of fungal growth with increased plant defence reaction during early colonization stages [78]. Further, the expression of host-specific resistance genes temporally differs significantly with the point of infection, which could add to the resistance in stepwise mode, i.e., at pathogen penetration and multiplication modes. The defence response in the resistant genotypes is associated with enhanced expression of *R* genes (*EcNBLRR83*: 3.07; *EcNBLRR55*: 2.94; *EcNBLRR30*: 2.42; *EcNBLRR32*: 2.02) during an early stage of pathogen infection leading to a hypersensitive reaction. On the other hand, the R genes differentially expressed in the contrasting genotypes VL Mandua-352 and Uduru Mallige in the late infection stage (*EcNBLRR76*: 2.45; *EcNBLRR45*: 2.31; *EcNBLRR26*: 2.13; *EcNBLRR23*: 2.11; *EcNBLRR21*: 2.06) are probably involved in the post-penetration multiplication of pathogen [79]. Interestingly, the early responsive *EcNBLRR83, EcNBLRR55, EcNBLRR30* and *EcNBLRR32* showed the *AS-1*, whereas the late responsive *EcNBLRRs* (*EcNBLRR76, EcNBLRR45*, *EcNBLRR26*, *EcNBLRR23* and *EcNBLRR21*) showed the *W-box* as common pathogen-inducible cis-regulatory elements in their promoter sequences. The overexpression of Benzothiadiazole and salicylic acid inducible *W-box* binding TF WRKY45 in Rice significantly enhanced resistance to rice blast fungus [76]. Furthermore, the binding of TGA family bZIP transcription factors to the as-1 element (TGACG) results in salicylic acid (SA)-mediated systemic acquired resistance [80]. Further the major portion (67%) of candidate genes showed the CC-NB-LRR domain structures. The CC–NB–LRR sub-class of NBLRRs is well represented in the Poaceae lineage. Several cloned genes in wheat for rust and powdery mildew genes are belonging to CC-NB-LRR sub-class [39]. The interactions of CC region with WRKY transcription factors in the nucleus reduce the WRKY factors ability to repress the *R*-gene expression [81]. Additionally, the CC domain of RPS5 is both necessary and sufficient for binding to protein kinase PBS1. However, the truncated form of RPS5 lacking the LRR domain requires the CC domain for the activity. Thus, the CC domain of NBLRRs may be involved in both detection of the pathogen signal and activation of the downstream response [82, 83].

## Conclusion

The current study identified 116 *EcNBLRR* genes through genome-wide mining with homology-based BLAST with query NBLRR sequences and HMM-search and HMM-scan with PFAM database. We also explored various structural characteristics features like motif analysis, subcellular prediction, and functional analysis for 116 identified *EcNBLRR* genes. The *EcNBLRR* genes have a broad range of variations in length, number of exons and intronic phases, molecular weight, isoelectric point and subcellular localization. *Cis*-acting analysis revealed various hormonal and light signalling and stress-responsive elements. Further, the lineage-specific gene loss events predominantly shaped the divergence of NBLRRs in the Gramineae species. The qRT-PCR gene expression analysis showed the association of nine *EcNBLRR* candidate genes *M. grisea* resistance reaction in resistant genotype VL Mandua-352. The present study generated the fundamental finger millet molecular breeding resource for enhancing blast resistance. The identified *EcNBLRRs* could be expanded to study their response to other diseases of finger millet. Furthermore, novel genome editing approaches could be employed for in-depth functional analysis and regulation of *EcNBLRRs* and their regulatory elements expression. The current *EcNBLRRS* also helps to generate and validate the R-gene-specific molecular markers and their subsequent utilization in the finger the millet molecular breeding programmes.

## Materials and methods

### Genome-wide mining, physicochemical characterization and chromosomal localization of NBLRR genes and proteins

The 88 and 126 *NBLRR* proteins of *Arabidopsis thaliana* and rice, respectively, were retrieved from TAIR (http://www.arabidopsis.org/) and the rice genome annotation project (http://rice.uga.edu) using search terms ‘NBSLRR’ and ‘NBLRR’. The latest target proteomes of finger millet (Ecorocona_560_v1.1.protein.fa), rice (Osativa_323_v7.0), sorghum (Sbicolor_730_v5.1), foxtail millet (Sitalica_312_v2.2.) and model grass plant purple false brome (Bdistachyon_556_v3.2) were downloaded from Phytozome (https://phytozome-next.jgi.doe.gov/). In the first step, the *A. thaliana* and rice query sequences were BLAST aligned against target proteomes with an e-value cut-off of *1e-5*. Secondly, the HMM models were separately built for *Arabidopsis thaliana* and rice *NBLRR* query protein sequences and the NB-ARC Pfam domain (PF00931; https://www.ebi.ac.uk/interpro/download/pfam/) were employed to perform the HMM search (e-value < *0.01*) with the target proteomes. The non-redundant hits from the first and second steps were subjected to an HMM-scan with the Pfam-A database (https://www.ebi.ac.uk/interpro/download/pfam/; e-value: 1e-3). The sequences showing the NBS domain towards the N (amino) terminal and atleast one LRR domain towards the C-terminal were considered for subsequent analysis (Additional File 15). Based on chromosomal positions the gene model hits were sequentially named based on chromosomal positions, and physicochemical properties of EcNBLRRs were predicted using the ProtParam tool (https://web.expasy.org/protparam). The subcellular localization was determined using the WoLFPSORT: Protein Subcellular Localization Prediction (https://wolfpsort.hgc.jp) server. Further, the identified EcNBLRRs were HMM-serched with TIR (PF01582) and RPW8 (PF05659) using HMM-Search with an e-value of 0.001. The coiled-coils (CC) towards N-terminals of EcNBLRRs were checked using Marcoil integrated in MPI Bioinformatics Toolkit (https://toolkit.tuebingen.mpg.de/tools/marcoil) with probability score of 0.4-1 [84].

### Domain, motifs, and gene structure analysis of ***NBLRR*proteins in finger millet**

The domain features of EcNBLRR proteins were examined using Pfam (https://www.ebi.ac.uk/interpro/download/pfam/; e-value: < *1e-3)* and SMART (https://smart.embl-heidelberg.de/) database with default parameters. The top ten conserved motifs of EcNBLRR proteins were predicted through MEME suit (https://meme-suite.org/meme) with default parameters [85]. The gene structure and intronic phases of *EcNBLRR* genes were visualized in TBtool [86] using the GFF3 file of *Eleusine coracana* downloaded from the Phytozome (https://phytozome-next.jgi.doe.gov/) database.

### ***Cis***-acting element analysis

The 1.5 kb upstream promoter region of each *EcNBLRR* gene from the start codon was examined for *cis*-acting elements with the PlantCare server (http://bioinformatics.psb.ugent.be/webtools/plantcare/html) [87]. The predicted *cis*-acting elements were classified into functional subcategories, and the distribution was visualized within the 1.5 kb upstream regions of *EcNBLRR* genes.

### Sequence alignment and phylogenetic analysis

For evolutionary analysis, the NBLRR homologs among the five Gramineae members *viz*., *B. distachyon, E. coracana, O. sativa, S. bicolor* and *S. itlaica*. The phylogenetic analysis was used to determine the evolutionary association among *NBLRR* sequences of finger millet with other target species. The multiple sequence alignment of *NBLRRs* was carried out using the MUSCLE [88]. The multiple sequence alignment was trimmed using the trimAL tool (http://trimal.cgenomics.org/) [89]. Then, the aligned and trimmed sequences were used for phylogenetic tree construction using IQTREE.v.16.12 (http://www.iqtree.org/) with the Maximum Likelihood Method and 1000 bootstrap replicates. The phylogenetic tree was visualized with iTOL v6 (https://itol.embl.de) server.

### Duplication, orthology, synteny and selection pressure analyses

The self-BLASTp search (e-value < *1e-5*) within the finger millet proteome was performed using BLAST + 2.12.0. Further, the DupGen_finder was used to analyse various duplications [90]. The orthologs among the *NBLRRs* of all the five target species were deciphered with OrthoFinder (https://github.com/davidemms/OrthoFinder) [91]. The ParaAT2.0 (https://ngdc.cncb.ac.cn/tools/paraat) software was used to align the *NBLRR* homolog pairs [92] and aligned homologs were used to calculate the non-synonymous (Ka), and synonymous rate (Ks). Subsequently, the evolutionary constraint of Ka and Ks among each *NBLRR* homolog pairs was calculated using KaKs calculator 3.0 with the GY-HKY method [93]. The whole proteome of finger millet was aligned with the whole proteomes of rice, sorghum, foxtail millet and purple false brome using the BLASTp program (*e-*value < *1e-5*). The MCScanX program was employed to identify the collinear blocks [94]. Further, the NBLRR gain and losses in the evolutionary lineage of the target species were worked with Notung-2.9 [95, 96].

### Plant material, pathogen inoculation, blast phenotyping and sample collection

The virulent finger millet *M. grisea* isolate Ragi Almora (FMg_Al) was used in this study to inoculate on resistant (VL Mandua-352) and susceptible (Uduru Mallige) genotypes under artificial conditions [97]. The fungal isolate was retrieved from preservation stock, multiplied on oatmeal agar medium (HiMedia Laboratory Pvt. Limited Bombay, India) and incubated at 26 ℃ with 12 h light and dark alternation periods [98]. The plants were grown under a controlled environment at a blast phenotyping facility of ICAR-IARI, New Delhi in a randomized complete block design (RCBD) with three replications. Each replicating plastic pot (9.5 × 10 cm) carried approximately fifty plants. After ten days of fungus incubation, the mycelium was gently scrapped using a sterile glass slide containing 10 ml of sterile water and filtered through a double-layered sterilized muslin cloth. Finally, culture suspension of *M. grisea* was adjusted to 1 × 10^6^ conidia/ml and added with 0.05% Tween 20 (HiMedia Laboratory Pvt. Limited Bombay, India) before inoculation on 20-day-old plants. The inoculated plants were maintained at 25 ± 1℃ and 95% RH with 12 h alternate photoperiods for blast development [97, 98]. The similar inoculation with sterile water alone served as a control. Leaf blast symptoms were calculated at 7–8 dpi (days post inoculation) using a 0–9 disease scale grade suggested by Babu and co-workers [98] (Additional File 16). The blast disease severity was assessed and expressed as per cent disease incidence (PDI) [97].

### RNA isolation, cDNA synthesis and qRT-PCR expression study

The total RNA was isolated from the replicated biological and technical samples from 0, 24 and 72 h intervals using Trizol reagent, Invitrogen, USA [97]. RNA quality and integrity were quantified in NanoDrop (Thermo Fisher Scientific, Waltham, Massachusetts, USA) and visualized in 1.5% aarose gel electrophoresis. After that, RNA was converted into cDNA (complementary-DNA) using a cDNA synthesis kit (Thermo Fisher Scientific, Waltham, USA). Twenty-two PCR primers were designed for selected *EcNBLRR* genes using primer3blast software (https://primer3plus.com/cgi-bin/dev/primer3plus.cgi) (Additional File 17). For qRT-PCR analysis, the first-strand cDNA was used (Roche Life Science, Penzberg, Germany) with finger millet endogenous coding gene *Actin* as internal control with the following conditions: denaturation at 95℃/2 min (initial), annealing of 35 cycles at 58℃/1.0 min and extension at 72℃/10s followed by three-step melting at 95℃/10s, 63℃/60s, and 97℃/10s, and cooling at 37℃/30s. The Ct mean values were considered for calculating 2^−∆∆CT^ [83], and fold change expression changes were estimated and interpreted [97, 99]. Two-way ANOVA was performed using the Bonferroni post-hoc test to determine the statistical significance.

### Electronic supplementary material

Below is the link to the electronic supplementary material.

**Additional File 1.** List of mined *NBLRR* genes in the finger millet genome and their physical locations. Note: bp- base pairs. (+) plus -sense strand, minus (-) -antisense strand

**Additional File 2.** List of mined NBLRR genes in the foxtail millet, purple false brome, rice and sorghum genomes for evolutionary analysis and their physical locations details. Note: bp- base pairs

**Additional File 3**. The detailed description of mined EcNBLRRs showing physical location and physiochemical properties of protein sequences. Note: aa- amino acids, bp-base pairs, MW-molecular weight, *pI*- isoelectric point

**Additional File 4.** The statistical significance and signatures of the best ten motifs identified in the 116 EcNBLRRs employing MEME server

**Additional File 5**. The rooted phylogenetic tree of 957 NBLRRs from four crop species, *viz.,* Finger millet, Rice, Sorghum, Foxtail millet and a model grass Purple false brome in a high-resolution image.The clusters I, II, III, IV, V, VI and VII are represented by dark red, dark orange, dark green, light green, loyal blue, purple and blue colours, respectively. The genes of Finger millet, Rice, Sorghum, Foxtail millet and Purple false brome are mentioned in dark green, dark red, orange, purple and blue colours, respectively

**Additional File 6**. The detailed descriptions on synonymous (Ks) and non-synonymous (Ka) substitutions among the NBLRR paralogs of finger millet. (Note- Ka: Nonsynonymous substitution rate, Ks: Synonymous substitution rate, Ka/Ks: Selective strength, P-Value(Fisher): The value computed by Fisher exact test, Length: Sequence length (after removing gaps and stop codon(s)), Substitutions: Substitutions between sequences, S-Substitutions: Synonymous substitutions and N-Substitutions: Nonsynonymous substitutions)

**Additional File 7.** The details on synonymous (Ks) and non-synonymous (Ka) substitutions among the NBLRR orthologs of finger millet, rice, purple false brome, sorghum and foxtail millet. (Note- Ka: Nonsynonymous substitution rate, Ks: Synonymous substitution rate, Ka/Ks: Selective strength, P-value(Fisher): The value computed by Fisher exact test, Length: Sequence length (after removing gaps and stop codon(s)), Substitutions: Substitutions between sequences, S-Substitutions: Synonymous substitutions and N-Substitutions: Nonsynonymous substitutions)

**Additional File 8**. The synteny and collinear association between the finger millet and other target species, *viz*., purple false brome, rice, sorghum and foxtail millet

**Additional File 9.** The arrangement and distribution of core cis-acting elements identified in 1.5kb upstream regions of NBLRR genes in finger millet

**Additional File 10.** The arrangement and distribution of growth and development related cis-acting elements identified in 1.5kb upstream regions of NBLRR genes in finger millet

**Additional File 11.** The arrangement and distribution of hormonal responsive cis-acting elements identified in 1.5kb upstream regions of NBLRR genes in finger millet

**Additional File 12.** The arrangement and distribution of light responsive cis-acting elements identified in 1.5kb upstream regions of NBLRR genes in finger millet

**Additional File 13.** The arrangement and distribution of stress responsive *cis*-acting elements identified in 1.5kb upstream regions of *NBLRR* genes in Finger millet

**Additional File 14.** (A) *M. grisea* isolate Ragi Almora (FMg_Al) multiplied on Oat meal agar; (B) Finger millet cultivars Uduru Mallige (susceptible) before inoculation of *M. grisea* isolate Ragi Almora (FMg_Al); (C) Finger millet cultivar VL Mandua-352 (resistant) before inoculation of *M. grisea* isolate Ragi Almora (FMg_Al); (D) Typical blast symptoms with higher blast on Uduru Mallige (PDI 82.59%) cultivar at 8 dpi; (E) Minute brown spots (hypersensitive reaction) on resistant cultivar VL Mandua-352 at 8 dpi (PDI 11.85%); (F) The close-up view of severe blast symptom observed on susceptible cultivar Uduru Mallige

**Additional File 15.** The pipeline employed to mine NBLRRs in finger millet and other target species

**Additional File 16.** The blast phenotyping 0–9 standard disease scale employed to phenotype blast incidence on finger millet genotypes

**Additional File 17.** The detailed description of qRT-PCR primers used to investigate the selected EcNBLRRs expressions at transcript level upon *M. grisea *infection

## Data Availability

All the raw data sets used in this study were downloaded from publicly available databases. The necessary supporting data is provided as a supplementary file.
