# Supplementary material for: Genome-wide identification and characterization of NBLRR genes in finger millet (Eleusine coracana L.) and their expression in response to Magnaporthe grisea infection
Source: BMC Plant Biol. 2024 Jan 29;24:75. doi: 10.1186/s12870-024-04743-z (PMC10823742; doi:10.1186/s12870-024-04743-z)
Supplement: Supplementary file 4 — Additional File 4. The statistical significance and signatures of the best ten motifs identified in the 116 EcNBLRRs employing MEME server [file 12870_2024_4743_MOESM4_ESM.pdf]

Motif Number and Signature		p-Value
Motifs-1	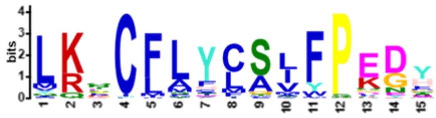	1.3e-704
Motifs-2	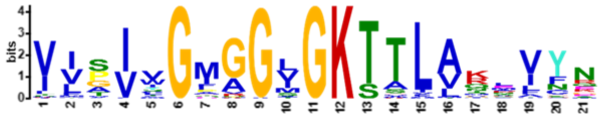	3.5e-869
Motifs-3	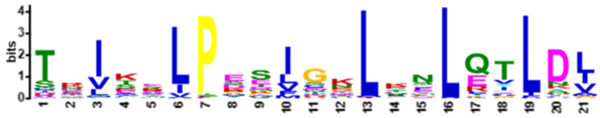	7.9e-653
Motifs-4	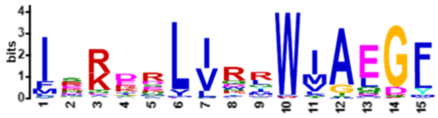	4.0e-544
Motifs-5	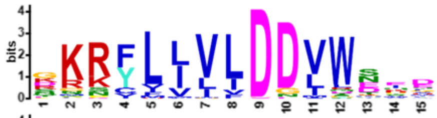	1.5e-458
Motifs-6	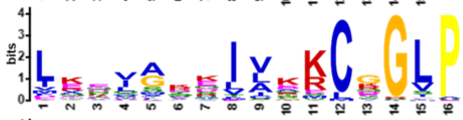	1.4e-428
Motifs-7	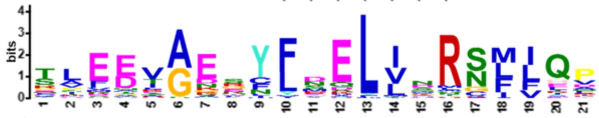	6.8e-474
Motifs-8	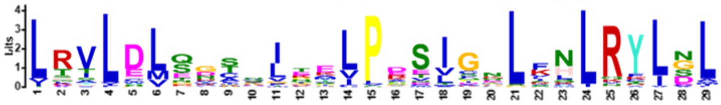	7.1e-355
Motifs-9	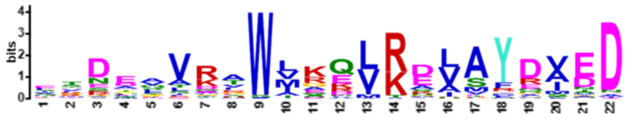	7.2e-451
Motifs-10	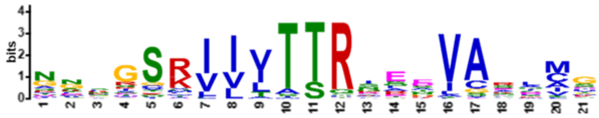	3.2e-451

Additional File 4. The statistical significance and signatures of the best ten motifs identified in the 116 EcNBLRRs employing MEME server.
