# Supplementary figures and images for: Genome-wide identification and characterization of NBLRR genes in finger millet (Eleusine coracana L.) and their expression in response to Magnaporthe grisea infection
Source: BMC Plant Biol. 2024 Jan 29;24:75. doi: 10.1186/s12870-024-04743-z (PMC10823742; doi:10.1186/s12870-024-04743-z)

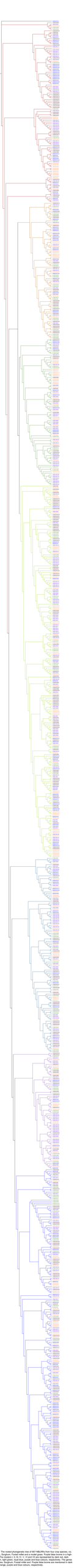

Supplement: Supplementary file 5 — Additional File 5. The rooted phylogenetic tree of 957 NBLRRs from four crop species, viz., Finger millet, Rice, Sorghum, Foxtail millet and a model grass Purple false brome in a high-resolution image.The clusters I, II, III, IV, V, VI and VII are represented by dark red, dark orange, dark green, light green, loyal blue, purple and blue colours, respectively. The genes of Finger millet, Rice, Sorghum, Foxtail millet and Purple false brome are mentioned in dark green, dark red, orange, purple and blue colours, respectively [file 12870_2024_4743_MOESM5_ESM.pdf]
