# Supplementary material for: Genome-wide identification and characterization of NBLRR genes in finger millet (Eleusine coracana L.) and their expression in response to Magnaporthe grisea infection
Source: BMC Plant Biol. 2024 Jan 29;24:75. doi: 10.1186/s12870-024-04743-z (PMC10823742; doi:10.1186/s12870-024-04743-z)
Supplement: Supplementary file 12 — Additional File 12. The arrangement and distribution of light responsive cis-acting elements identified in 1.5kb upstream regions of NBLRR genes in finger millet [file 12870_2024_4743_MOESM12_ESM.pdf]

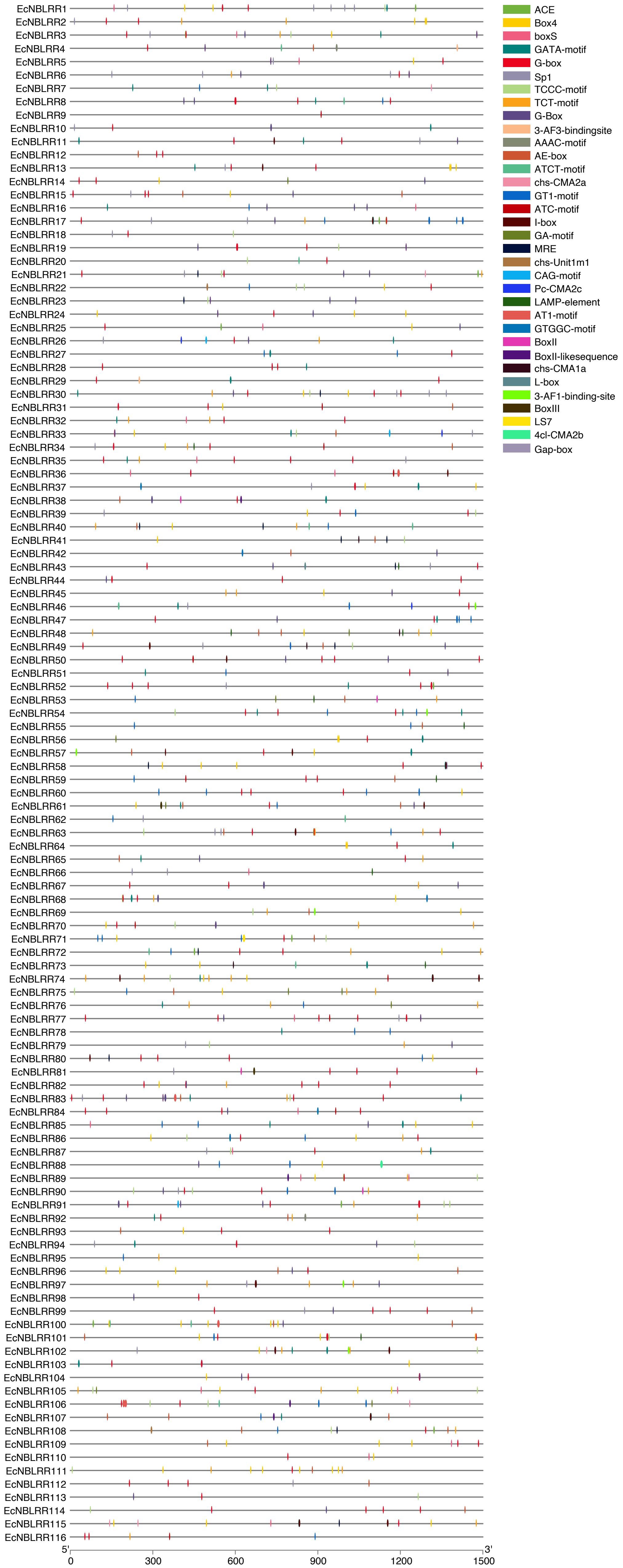

Additional File 12. The arrangement and distribution of light responsive cis-acting elements identified in 1.5kb upstream regions of NBLRR genes in finger millet
