# Supplementary material for: Genome-wide identification and characterization of NBLRR genes in finger millet (Eleusine coracana L.) and their expression in response to Magnaporthe grisea infection
Source: BMC Plant Biol. 2024 Jan 29;24:75. doi: 10.1186/s12870-024-04743-z (PMC10823742; doi:10.1186/s12870-024-04743-z)
Supplement: Supplementary file 13 — Additional File 13. The arrangement and distribution of stress responsive cis-acting elements identified in 1.5kb upstream regions of NBLRR genes in Finger millet [file 12870_2024_4743_MOESM13_ESM.pdf]

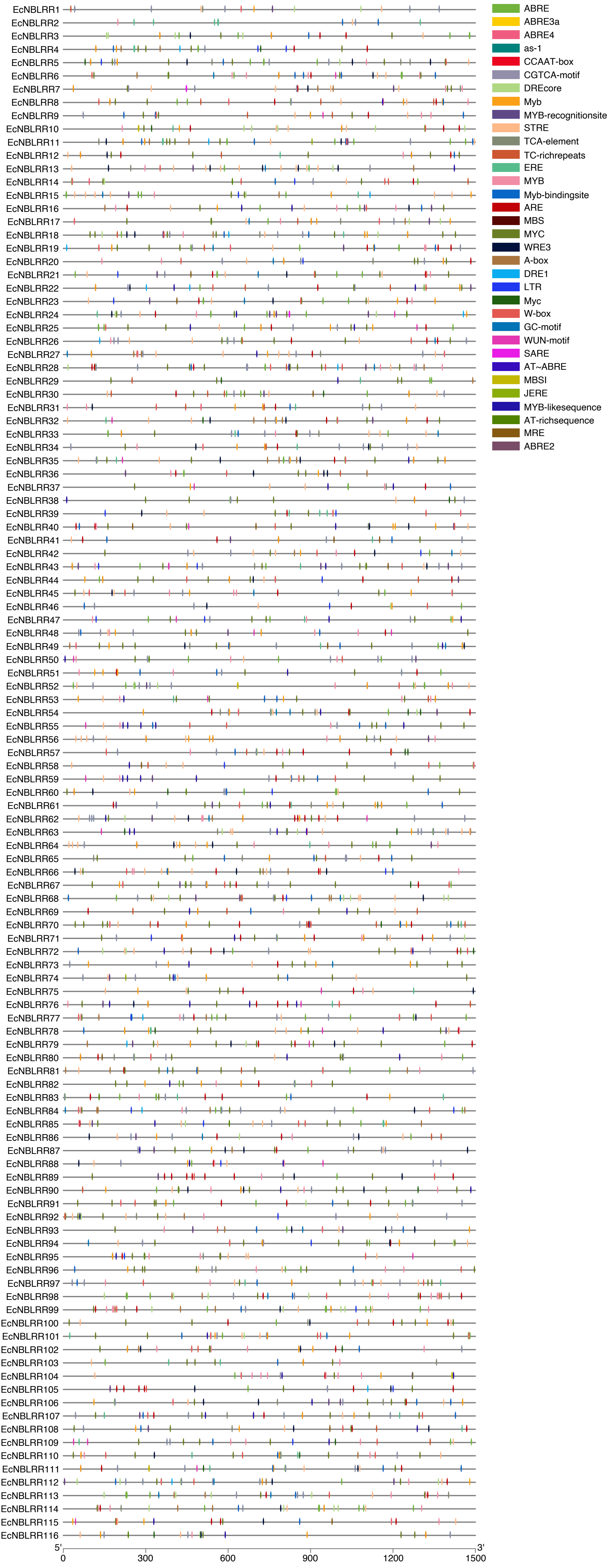

Additional File 13. The arrangement and distribution of stress responsive *cis*-acting elements identified in 1.5kb upstream regions of *NBLRR* genes in Finger millet.
