# Supplementary material for: Genome-wide identification and characterization of NBLRR genes in finger millet (Eleusine coracana L.) and their expression in response to Magnaporthe grisea infection
Source: BMC Plant Biol. 2024 Jan 29;24:75. doi: 10.1186/s12870-024-04743-z (PMC10823742; doi:10.1186/s12870-024-04743-z)
Supplement: Supplementary file 14 — Additional File 14. (A) M. grisea isolate Ragi Almora (FMg_Al) multiplied on Oat meal agar; (B) Finger millet cultivars Uduru Mallige (susceptible) before inoculation of M. grisea isolate Ragi Almora (FMg_Al); (C) Finger millet cultivar VL Mandua-352 (resistant) before inoculation of M. grisea isolate Ragi Almora (FMg_Al); (D) Typical blast symptoms with higher blast on Uduru Mallige (PDI 82.59%) cultivar at 8 dpi; (E) Minute brown spots (hypersensitive reaction) on resistant cultivar VL Mandua-352 at 8 dpi (PDI 11.85%); (F) The close-up view of severe blast symptom observed on susceptible cultivar Uduru Mallige [file 12870_2024_4743_MOESM14_ESM.pdf]

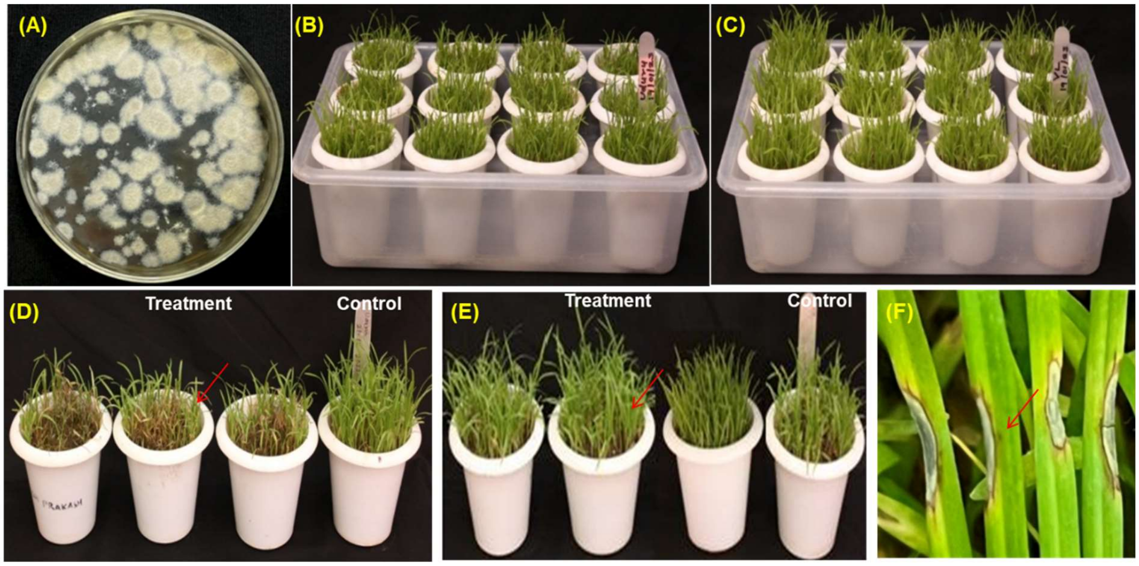


**Additional File 14.** (A) *M. grisea* isolate Ragi Almora multiplied on Oatmeal agar; (B) Finger millet cultivars Uduru Mallige (susceptible) before inoculation of *M. grisea* isolate Ragi Almora; (C) Finger millet cultivar VL Mandua-352 (resistant) before inoculation of *M. grisea* isolate Ragi Almora; (D) Typical blast symptoms with higher blast on Uduru Mallige (PDI 82.59 %) cultivar at 8 dpi; (E) Minute brown spots (hypersensitive reaction) on resistant cultivar VL Mandua-352 at 8 dpi (PDI 11.85 %); (F) The close-up view of severe blast symptom observed on susceptible cultivar Uduru Mallige.
