# Supplementary material for: Genome-wide identification and characterization of NBLRR genes in finger millet (Eleusine coracana L.) and their expression in response to Magnaporthe grisea infection
Source: BMC Plant Biol. 2024 Jan 29;24:75. doi: 10.1186/s12870-024-04743-z (PMC10823742; doi:10.1186/s12870-024-04743-z)
Supplement: Supplementary file 15 — Additional File 15. The pipeline employed to mine NBLRRs in finger millet and other target species [file 12870_2024_4743_MOESM15_ESM.pdf]

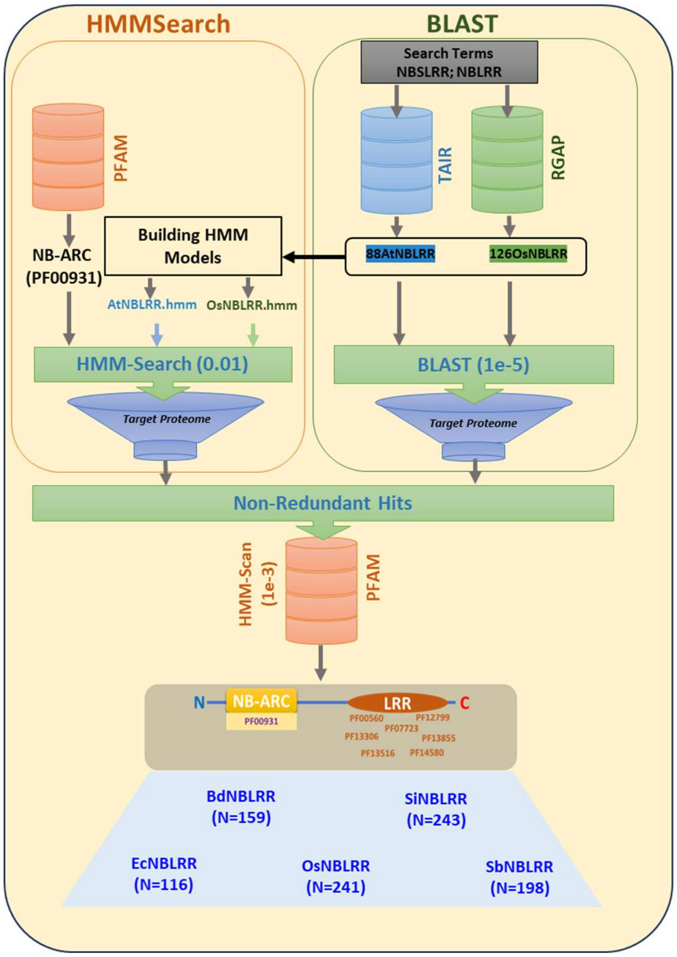

Proteome Versions of Target Species

- Finger millet: Ecorocona_560_v1.1
- Rice: Osativa_323_v7.0
- Sorghum: Sbicolor_730_v5.1
- Foxtail millet: Sitalica_312_v2.2.
- Purple false brome: Bdistachyon_556_v3.2

TAIR: https://www.arabidopsis.org/
RGAP: http://rice.uga.edu/

Additional File 15. The pipeline employed to mine NBLRRs in Finger millet and other target species.
